# Experimental Models to Study the Pathogenesis of Malaria-Associated Acute Respiratory Distress Syndrome

**DOI:** 10.3389/fcimb.2022.899581

**Published:** 2022-05-23

**Authors:** Samantha Yee Teng Nguee, José Wandilson Barboza Duarte Júnior, Sabrina Epiphanio, Laurent Rénia, Carla Claser

**Affiliations:** ^1^ A*STAR Infectious Diseases Labs (A*STAR ID Labs), Agency for Science, Technology and Research (A*STAR), Singapore, Singapore; ^2^ Department of Parasitology, Institute of Biomedical Sciences, University of São Paulo, São Paulo, Brazil; ^3^ Department of Clinical and Toxicological Analyses, Faculty of Pharmaceutical Science, University of São Paulo, São Paulo, Brazil; ^4^ Lee Kong Chian School of Medicine, Nanyang Technological University, Singapore, Singapore; ^5^ School of Biological Sciences, Nanyang Technological University, Singapore, Singapore

**Keywords:** *Plasmodium berghei*, mouse, ARDS, pulmonary vascular damage, endothelial dysfunction, vascular permeability

## Abstract

Malaria-associated acute respiratory distress syndrome (MA-ARDS) is increasingly gaining recognition as a severe malaria complication because of poor prognostic outcomes, high lethality rate, and limited therapeutic interventions. Unfortunately, invasive clinical studies are challenging to conduct and yields insufficient mechanistic insights. These limitations have led to the development of suitable MA-ARDS experimental mouse models. In patients and mice, MA-ARDS is characterized by edematous lung, along with marked infiltration of inflammatory cells and damage of the alveolar-capillary barriers. Although, the pathogenic pathways have yet to be fully understood, the use of different experimental mouse models is fundamental in the identification of mediators of pulmonary vascular damage. In this review, we discuss the current knowledge on endothelial activation, leukocyte recruitment, leukocyte induced-endothelial dysfunction, and other important findings, to better understand the pathogenesis pathways leading to endothelial pulmonary barrier lesions and increased vascular permeability. We also discuss how the advances in imaging techniques can contribute to a better understanding of the lung lesions induced during MA-ARDS, and how it could aid to monitor MA-ARDS severity.

## 1 Introduction

Malaria is a fatal parasitic disease that continues to threaten many human populations, especially in the tropical and subtropical regions of the world. Globally, the disease incidences and death rates have been declining slowly since 2000, as a result of numerous global initiatives. Nevertheless, in 2020, there were an estimated 241 million malaria cases with 627 000 people dying from the disease (WHO, 2021). Mortality from severe malaria remains a significant concern where increasing deaths were reported in South Asia, South America, and Eastern Mediterranean regions, apart from the African continent still accounting for most of the global deaths. Among the nine clinically important *Plasmodium* parasite species responsible for the disease in humans, *P. vivax* and mostly *P. falciparum* are the main causes of severe disease and death (Price et al., 2007; Olliaro, 2008). The other *Plasmodium* species includes *P. ovale*, *P. malariae*, *P. knowlesi*, *P. cynomolgi*, *P. brasilianum*, *P. simium* and *P. inui*, where the last five are zoonotic infections. Despite increased in detection, infection by *P. brasilianum, P. simium and P. inui* are still rare (Singh et al., 2004; Ta et al., 2014; Lalremruata et al., 2015; Brasil et al., 2017; Liew et al., 2021).

The clinical manifestations of malaria and severity are dependent on the host’s immune status, infection history, parasite virulence, and genetic variations in both the host and parasite. The first clinical symptoms of malaria are mostly non-specific including flu-like symptoms, fever paroxysm, headache, chills, weakness, myalgias, diarrhea, and nausea (Karunaweera et al., 2003; Bartoloni and Zammarchi, 2012). A large majority of *Plasmodium* infections are uncomplicated and rapidly resolve with early diagnosis and prompt treatment with relevant anti-malarials combinations (van der Pluijm et al., 2021). However, delayed diagnosis or inability to clear the blood forms of the parasite, particularly dire for the virulent *falciparum* malaria, predisposes infected individuals to severe clinical manifestations.

## 2 MA-ARDS in Humans

Malaria-associated acute respiratory distress syndrome (ARDS) is a complication of severe malaria that is less characterized compared to the most lethal cerebral malaria. MA-ARDS has been described to occur mainly in adults during infection by most clinically important *Plasmodium* species, including *P. knowlesi*, *P. ovale* and *P. malariae* (Lozano et al., 1983; Daneshvar et al., 2009; Haydoura et al., 2011; Taylor et al., 2012). MA-ARDS arising from severe *P. falciparum* and severe *P. vivax* single-infections in adults are more common, with varying prevalence of 5 - 25%, and 1 - 22% respectively (Mohan et al., 2008; Taylor et al., 2012; Val et al., 2017). In recent years, the rising incidence of adult MA-ARDS in Southeast Asia (particularly Malaysia), were largely attributed to severe zoonotic *P. knowlesi* infection, with a prevalence of 6 - 23% (Daneshvar et al., 2009; William et al., 2011; Barber et al., 2013). With the less frequently observed severe *P. ovale* infection, MA-ARDS prevalence was about 22% (Groger et al., 2017). Pregnant women with severe *falciparum* malaria, are predisposed to develop MA-ARDS and have an estimated prevalence of 29% (Taylor et al., 2012). MA-ARDS in adults is a fatal lung complication with a high fatality rate of at least 50% (Van den Steen et al., 2013; Val et al., 2017). On the other hand, MA-ARDS occurrence is rare in infected children. Respiratory distress arising in this population is predominantly caused by lactic acidosis, and to a certain extent by cardiac failure and cerebral complications (Taylor et al., 1993; Krishna et al., 1994; Dzeing-Ella et al., 2005; Taylor et al., 2012). Mortality from MA-ARDS is greatly dependent on several risk factors such as the virulence of *Plasmodium* species, severity of disease upon admission, immune and age status, pre-existing comorbidities, infections or disorders, timing and effectiveness of treatments administered, and availability of respiratory management therapies.

Uncomplicated malaria patients with probable respiratory distress may present early signs of a self-limiting cough with or without expectoration, tachypnea and tachycardia or appear asymptomatic (Maguire et al., 2005; Taylor et al., 2012). Moreover, respiratory distress symptoms may develop suddenly or exacerbate into MA-ARDS after several hours to days of well-tolerated anti-malarial treatment. This is despite diminishing or negative parasitemia (as assessed by microscopy) (Tong et al., 1972; Maguire et al., 2005; Taylor et al., 2012; Elzein et al., 2017), as frequently reported for *vivax* malaria patients receiving standard antimalarial treatments (Anstey et al., 2007; Val et al., 2017). Given that *P. vivax* infection results in a low parasitemia, this suggests that *P. vivax* and its by-products possess greater inflammatory potential that can trigger MA-ARDS post-antimalarial treatment (Karunaweera et al., 1992). Currently, there are no specific treatments for MA-ARDS, only respiratory management interventions following non-malaria ALI/ARDS therapeutic guidelines are implemented.

Patients with MA-ARDS develop clinical edema and diminished lung function (i.e., reduced gas transfer and small airway obstruction) (Anstey et al., 2002; Maguire et al., 2005). The histopathological hallmarks of MA-ARDS observed from human post-mortem lung sections were non-cardiogenic pulmonary edema, inflammation, hemorrhages, and alveolar damage (Taylor et al., 2012). Pulmonary edema is described as the flooding of the alveolar and/or interstitium with proteinaceous fluid. Diffuse alveolar damage is characterized by thickened alveolar septa, that may be plastered by eosinophilic hyaline membranes. Pulmonary inflammation is observed as the congestion of the alveolar capillaries with mono- and polymorphonuclear cells and/or infiltration of these leukocytes into the alveolar or interstitial space. Malaria pigments or hemozoins (Hz), either free or phagocytosed were found to be present as well. Immunostaining of the lung sections revealed that infiltrating nucleated cells were mostly CD3^+^T lymphocytes, CD68^+^ monocytes/macrophages, and to a lower extent, neutrophils (Valecha et al., 2009; Lacerda et al., 2012; Milner et al., 2013).

Most pathophysiological characterization of clinical MA-ARDS were drawn from autopsy lung sections, and these are potentially confounded by biasness from snapshot sampling and post-mortem artifacts. Therefore, attempts to study the disease progression in living MA-ARDS patients have been done by assessing the lung function with non-invasive methods. In one study, using a portable pulmonary testing station (TT544; Morgan Transflow System; Morgan Medical) where gas transfer and spirometry (breathing test) were assessed, patients were found to have impaired alveolar capillary membranes, resulting in edema and decreased blood volume in the pulmonary capillaries. The authors have suggested the occlusion of the pulmonary capillaries by immune cells and sequestered infected red blood cells (iRBCs) (Maguire et al., 2005). Findings from this study corroborated with histopathological findings gathered from autopsy series; however, studies beyond these non-invasive tests to gain mechanistic insights of MA-ARDS have proven difficult in living patients.

## 3 MA-ARDS Animal Model

The pathophysiology of MA-ARDS is highly complex and cannot be fully elucidated by clinical observations of infected humans. Undeniably, these clinical studies have revealed the critical hallmarks and correlates of the pathogenesis, yet it deters further understanding of the mechanisms in pathogenesis. Therefore, animal models of MA-ARDS are essential to conduct mechanistic studies, discover potential interventions, and validate the hypotheses drawn from clinical studies. Several combinations of experimental animal host and *Plasmodium* parasites have been used to induce MA-ARDS were summarized. Details about each experimental animal model are listed in **Table 1**, including the parasite species, inoculation, and pathological findings in the lung.

**Table 1:** Animal models of MA-ARDS.

Animal	Strain	Parasite specie	Parasite strain/clone	Inoculation		First exhibition of lung pathology (days post infection)	Peripheral parasitemia (%)	Lung pathological findings	Other affected organ(s)	Ref
				Route	Dose					
Mouse	C57BL/6 C57BL/6N C57BL/6J	*P. berghei*	ANKA	IP	10^6^ iRBCs	6	>0.5%	(↑) Lung edema, Alveolar capillary permeability (Evans blue); Alveolar infiltration with mononuclear and PMN cells	Brain	(Chang et al., 2001)
				IP	10^5^ iRBCs	7-10	NR	(↑) Lung weight; Alveolar edema (proteinaceous); Alveolar capillary permeability (Evans blue); Airway reactivity; Alveolar and interstitial infiltration with leukocytes (macrophages, monocytes); Levels of TNF-α, IL-12, IL-1β, IL-6, MCP-1/CCL2, RANTES/CCL5, KC/CXCL1 in lung tissue homogenate	Brain	(de Azevedo-Quintanilha et al., 2016)
			ANKA Clone 15cy1	IP	10^6^ iRBCs	6	20	Thickened alveolar septa (↑) Alveolar edema; Paracellular fluid hyperpermeability; Alveolar infiltration with leukocytes, ROS production by iRBCs sequestered in the lung microvasculature	NR	(Anidi et al., 2013)
			ANKA (MR4)	IP	5×10^5^ iRBCs (Male) 10^6^ iRBCs (Female)	6	3-5	(↑) IgM in BALF; Alveolar edema (proteinaceous); Levels of IFN-γ, IL-6, MIP-2/CXCL2, KC/CXCL1 in lung tissue homogenate; Interstitial inflammation	Brain	(Lovegrove et al., 2008)
			ANKA GFP-*luciferase* Clone 15cy1	IP	10^5^-10^6^ iRBCs	7	NR	(↑) Parasite sequestration	Brain	(Franke-Fayard et al., 2010)
			ANKA GFP-*luciferase* Clone 231cl1	IP	10^6^ iRBCs	6-7	5	(↑) Lung edema (Bronchi opacity by MRI); Lung weight; Alveolar capillary permeability (Vascular leakage); Parasite sequestration; Lung infiltration of activated CD8^+^ T cells, MODC, MDM (↓) Tight junction protein ZO-1 expression on lung epithelium; Tissue infiltration with cDCs, pDCs, macrophages	Brain	(Claser et al., 2019)
	DBA/2		ANKA	IP	10^6^-10^7^ iRBCs	7-12	30-50	Thickened alveolar septa (↑) Enhanced respiratory pause (Penh); Alveolar capillary permeability (Evans blue); Alveolar and interstitial infiltration of neutrophils, macrophages; Levels of VEGF, VEGF receptor (sFLT1) in serum and lung homogenate (↓) Respiratory frequency	NR	(Epiphanio et al., 2010)
			ANKA Clone 1.49L	IP	10^6^ iRBCs	7	15	Hydrothorax (↑) Penh; Parasite sequestration; Alveolar infiltration of neutrophils, leukocytes; Levels of myeloperoxidase in the BALF and lung homogenate; Levels of KC/CXCL1, MIP-2/CXCL2 in serum	NR	(Sercundes et al., 2016)
								Damaged alveolar septa; Eosinophilic/hyaline membranes adhered to the alveolar ducts and walls (↑) Lung weight; Pleural effusion; Haemorrhages; Alveolar edema; Alveolar capillary permeability (Evans blue); Alveolar and interstitial infiltration of neutrophils, macrophages, haemorrhages, Pulmonary opacification	NR	(Ortolan et al., 2014)
								Ground-glass opacification (↑) Penh; Expiration time; Pulmonary lesions (↓) Respiratory frequency, Tidal volume; Ventilation volume; Aerated tissue	NR	(Quirino et al., 2020)
	129P2Sv/Ev		ANKA Clone BdS	IP	10^6^ iRBCs	7-10	NR	(↑) Lung edema; Tissue infiltration with leukocytes (macrophages, neutrophils, T cells); Levels of IFN-γ, TNF-α, MCP-1/CCL2, MIP-1α/CCL3, RANTES/CCL5, IP-10/CXCL10, CCR2, CCR5, CCR1, CXCR3 in lung tissue homogenate	Brain	(Belnoue et al., 2008)
	129SV/J		ANKA (MR4)	IP	5×10^5^ iRBCs (Male) 10^6^ iRBCs (Female)	6	9	(↑) IgM in BALF	NR	(Lovegrove et al., 2008)
	BALB/c		ANKA		10^5^-10^7^ iRBCs	6	6-10	Thickened alveolar septa (↑) IgM in BALF, Lung edema; Alveolar infiltration with leukocytes (macrophages, lymphocytes)	NR	(Hansen et al., 2005; Lovegrove et al., 2008; Epiphanio et al., 2010)
			ANKA Clone 15cy1	IP	10^4^ iRBCs; drinking water supplemented with 4-amino benzoic acid (0.375 mg/ml PABA)	10	10	(↑) Lung weight; IgM in BALF; Alveolar infiltration with leukocytes (macrophages, lymphocytes)	NR	(Vandermosten et al., 2018)
	CBA/J		ANKA	IP	10^6^ iRBCs	6-10	10	Neutrophils, monocytes-containing Hz, megakaryocytes adhered to the endothelium; Diffuse pulmonary and alveolar edema	Brain, liver	(Carvalho et al., 2000)
	ICR		ANKA	IP	2×10^7^ iRBCs	5	50	Congestion of iRBCs in the alveolar space; Hyaline membrane adhered to the alveolar wall; Pulmonary inflammation (↑) Levels of IL27 in serum	Liver	(Fazalul Rahiman et al., 2013)
	Swiss		ANKA	IP	10^7^ iRBCs	7	68	(↑) Lung weight; Haemorrhages	Liver, kidney	(Moore et al., 2008)
	C57BL/6 C57BL/6J		NK65	IP	10^4^ iRBCs; drinking water supplemented with 4-amino benzoic acid (0.375 mg/ml PABA)	9	5	Thickened alveolar septa (↑) Lung weight; Pleural effusion; Haemorrhages; Alveolar edema; Alveolar capillary permeability (Evans blue); Airway reactivity; Tissue infiltration with CD8^+^ T cells, MODC; Levels of IFN-γ, TNF-α, MCP-1/CCL2, MIP-1α/CCL3 in the lung tissue homogenate	NR	(Galvao-Filho et al., 2019)
			NK65	IP	10^4^ iRBCs; drinking water supplemented with 4-amino benzoic acid (0.375 mg/ml PABA)	8-12	5-20	Eosinophilic/hyaline membranes adhered in the alveolar space (↑) Lung weight; Pleural effusion; Haemorrhages; Alveolar and interstitial edema (Proteinaceous); Alveolar capillary permeability (Evans blue); Alveolar infiltration with leukocytes (macrophages-containing Hz, neutrophils, lymphocytes) and haemorrhages; Levels of IFN-γ, TNF-α, IL-10, IP-10/CXCL10, MIP-2/CXCL2, MCP-1/CCL2, KC/CXCL1, VEGF, PIGF in the lung tissue homogenate	NR	(Van den Steen et al., 2010)
			NK65 Clone 2168cl2	IP	10^4^ iRBCs; drinking water supplemented with 4-amino benzoic acid (0.375 mg/ml PABA)	7	6	(↑) Levels of PIGF, VEGF-A in BALF; Levels of PIGF, VEGF-A, TNF-α, IP-10/CXCL10, MIP-2/CXCL2 in the lung tissue homogenate; Tissue infiltration with CD8^+^ T cells;	NR	(Pham et al., 2017)
			NK65 GFP-*luciferase* E line (Clone 2168cl2)	IP	10^4^ iRBCs; drinking water supplemented with 4-amino benzoic acid (0.375 mg/ml PABA)	9	20	(↑) Alveolar edema (proteinaceous); IgM in BALF; Alveolar infiltration with leukocytes (macrophages, lymphocytes, neutrophils) and hemorrhages	NR	(Vandermosten et al., 2018)
	C3H/z		KEYBERG Clone 173	IP	3×10^6^ iRBCs	8	>6	Severe alveolar and interstitial edema (proteinaceous) (↑) Alveolar infiltration with macrophages	Heart	(Weiss and Kubat, 1983)
	C57BL/6	*P. chabaudi*	CB	IP	10^5^ iRBCs	9	50	(↑) IgM in BALF; Alveolar infiltration with leukocytes (myeloids, neutrophils, T cells) and Hz; Cell death of lung tissue; MRP14-associated neutrophil response; Levels of IFN-γ, IL-6, KC/CXCL1, LIX/CXCL5	NR	(Lin et al., 2017)

	BALB/c	*P. yoelii*	17XL	IP	2×10^5^ iRBCs	6	90	(↑) Alveolar and interstitial edema (proteinaceous); Alveolar infiltration of mononuclear and PMN cells	Liver, spleen, kidney	(Fu et al., 2012)
	BALB/c	*P. vinckei*	NR	IP	10^5^ iRBCs	NR	NR	(↑) Interstitial infiltration of mononuclear cells, neutrophils	Liver, kidney	(Kremsner et al., 1992)
Hamster	NR	*P. berghei*	NYU-2	NR	NR	6	NR	Protein-rich exudate in the alveolar space; Congested capillaries with presence of macrophages; Lymphatic thrombosis	NR	(MacCallum, 1968; MacCallum, 1969)
Non-human primate	*Macaca mulatta* (Rhesus macaque)	*P. knowlesi*	NR	NR	NR	6-7	>70	Mild lung edema; Congested vessels with fibrin deposit, thrombi, mononuclear and PMN cells	Kidney, lymph nodes	(Spangler et al., 1978)
	*Macaca fascicularis* (kra/cynomolgus macaques)		Clone Pk1(A+)	IV	10^5^ sporozoites	6-50	<1	Thickened interstitium; Haemorrhage; Fibrosis; Hyperplasia (↑) Parasite sequestration in the lung microvasculature	Liver, kidney, spleen	(Peterson et al., 2021)
	*Papio Anubis* (Olive baboon)		H strain	IV	10^4^-10^6^ iRBCs	12-28	5-50	Thickened alveolar septa (↑) Alveolar and interstitial edema; Alveolar infiltration with leukocytes (macrophages-containing Hz, neutrophils), Parasite sequestration	Brain, liver, kidney	(Ozwara et al., 2003)
	*Macaca mulatta*	*P. cynomolgi*	M/B strain	IV	2×10^3^ sporozoites	23	<20	Eosinophilic membranes adhered on the parenchyma; Fibrin deposits on interstitium; Thickened alveolar wall (↑) Alveolar and interstitial edema; Alveolar infiltration with macrophages-containing Hz	Liver, kidney, spleen	(Joyner et al., 2017)
	*Saimiri boliviensis* (Black-capped squirrel monkey) (splenectomised)	*P. vivax*	Brazil VII strain	IV	10^4^ sporozoites	10-25	<1	Thickened alveolar wall (↑) Interstitial edema; Alveolar infiltration with leukocytes (macrophages), Hz, haemorrhages; Parasite sequestration	Liver, kidney	(Peterson et al., 2019)
	*Macaca mulatta* (splenectomised)	*P. coatneyi*	NR	IV	NR	NR	NR	(↑) Parasite (schizonts) sequestration in the lung microvasculature	Liver, kidney, heart	(Desowitz et al., 1969)

IP, Intraperitoneal; IV, Intravenous; iRBCs, Infected red blood cells; GFP, Green fluorescent protein; BALF, Bronchoalveolar lavage fluid; TNF, Tumour necrosis factor; IFN, Interferon; IL, Interleukin; CXCL, CXC chemokine ligand; CCR, CC chemokine receptor; KC, Keratinocyte chemoattractant; MCP, Monocyte chemoattractant protein; MIP, Macrophage inflammatory protein; VEGF, Vascular endothelial growth factor; PIGF, Placental growth factor; PMN, Polymorphonuclear; MODCs, Monocyte-derived dendritic cells; MDMs, Monocyte-derived macrophages; Hz, Hemozoin; NR, Not reported; (↓), Decreased; (↑), Increase.

### 3.1 Rodent Models

The use of rodents as experimental animal hosts makes up most of the experimental models of MA-ARDS developed, due to the practical advantages it offers as a research tool including their small size, various well-established strains, ease of genetic modifications and controlled genetic background.

To date, various strains of mouse inoculated with any of the four rodent *Plasmodium* species, namely, *P. berghei*, *P. yoelii*, *P. chabaudi* and *P. vinckei*, can develop MA-ARDS with varying similarities to the human disease. The C57BL/6 mouse strain develops MA-ARDS when infected with the ANKA strain of *P. berghei* (PbA) (Bafort and Timperman, 1969) and has been used widely for many MA-ARDS studies. Apart from developing MA-ARDS, this is an established model for experimental cerebral malaria (ECM) where at least 60% of infected mice succumb to fulminant cerebral pathology followed by death (Engwerda et al., 2005) before lung pathology can be fully established or studied upon. Nevertheless, it has been demonstrated in this model that MA-ARDS occurs independently of ECM development (Hansen et al., 2005; Lovegrove et al., 2008; Claser et al., 2019). Yet, the lethal onset of cerebral complications in this model restricts the study of MA-ARDS to the early phase of disease development. In view of that, alternative mouse models of MA-ARDS resistant to cerebral complications have been developed to provide a broader window period to study the late phase of lung complication. Epiphanio et al. developed a model inoculating PbA into DBA/2 mouse strain resistant to ECM, where on average, 50% of infected mice develops MA-ARDS (Epiphanio et al., 2010; Sercundes et al., 2016; Dos Santos Ortolan et al., 2019). The infection of C57BL/6 mouse strain with the NK65 strain of *P. berghei* (PbNK65) which were resistant to ECM as well, induces a higher incidence of MA-ARDS (at least 90%) in infected mice (Van den Steen et al., 2010). Infection of C57BL/6 mouse strain with CB strain of *P. chabaudi* is another model of MA-ARDS reported to be resistant to cerebral complications (Lin et al., 2017).

Lung injuries sustained in mouse models of MA-ARDS parallels the clinical pathology. Histological lung sections of infected mice showed flooded alveolar space and interstitium with proteinaceous fluid also containing various inflammatory cells. The integrity of alveolar capillaries was disrupted and mostly leukocytes and iRBCs were often found congested within these capillaries. Lung function test coupled with sophisticated imaging procedures [i.e., X-ray, single-photon emission computed tomography/computed tomography (SPECT/CT) and magnetic resonance imaging (MRI)] conducted on conscious infected mice exhibited pulmonary aeration disturbance, reduced respiration frequency, obstructed airways and increasing timely pulmonary opacities (Ortolan et al., 2014; Claser et al., 2019; Quirino et al., 2020). Ultrastructural analysis using transmission electron microscopy (TEM) in the lung of PbA-infected DBA/2 mice that died of MA-ARDS, provided evidence of eosinophilic hyaline membranes occasionally found to line the alveolar septa (Aitken et al., 2014). However, it was highlighted in previous studies that in some parasite and mouse strain combinations, the lung pathology deviates from the MA-ARDS hallmarks reported in patients. *P. berghei* K173 infection of C57BL/6 mice results in non-proteinaceous edema restricted to the interstitium (Hee et al., 2011; Van den Steen et al., 2013). On the other hand, infection of C57BL/6 mice with the AS strain of *P. chabaudi* induced minimal edema (Van den Steen et al., 2010; Lin et al., 2017). These differences in lung pathology between the mouse models and human beings must be kept in mind before drawing definitive conclusions. Moreover, the details of disease progression, infection method, parasite inoculum dose, parasite line/clone and mouse genetic background should be deeply examined to select suitable mouse models of MA-ARDS and draw valid comparisons between mouse models.

### 3.2 Non-Human-Primate (NHP) Models

Although rodent models have helped to decipher MA-ARDS pathogenesis and evaluate potential therapeutics, it remains an open question about their full relevance to the human lung pathology. Therefore, NHP models may be more suited since they possess greater genomic, immunogenic, and physiological homologies to humans than rodents.

Some of the earliest reports of MA-ARDS in *Plasmodium*-infected NHP hosts dates as far back as 50 years. Primate malarial species, *P. coatneyi* or *P. knowlesi*, were experimentally inoculated into rhesus macaques (*Macaca mulatta*) and caused multiple organ damages, with gross findings of lung injury involving congested alveolar capillaries with iRBCs (Desowitz et al., 1969; Spangler et al., 1978). In recent years, there has been more detailed documentation of MA-ARDS in NHP hosts such as rhesus or cynomolgus macaques (*Macaca fascicularis*), and black-capped squirrel monkey (*Saimiri boliviensis*) experimentally infected with human-malaria causing *Plasmodia*, like *P. vivax, P. cynomolgi*, or *P. knowlesi*. In these studies, histopathological findings from lung sections of infected NHP parallels those obtained in mouse models and in humans (Joyner et al., 2017; Peterson et al., 2019; Peterson et al., 2021). However, aside from these histological findings, progress into understanding the pathophysiological processes in MA-ARDS with NHP models has remained limited.

However, one major caveat of the studies using NHP was that the animals were splenectomized to induce higher parasitemia and immunogenic responses. Splenectomy in NHP hosts may alter the course of disease progression, parasite clearance and parasite cytoadhesion and sequestration dynamics (David et al., 1983). In addition, NHP hosts used in these studies were rarely naïve as they are often re-employed as experimental animal after clearance of previous experimental infections (e.g., malaria or viral infections), or were pre-exposed to malaria, or other pathogens while in breeding colonies. The latter was reported in the study by Spangler et al. where NHP host was potentially infected by *Pneumonyssus simicola* (lung mite) (Spangler et al., 1978). Such pre-exposure to pathogens may have an immune-modulating effect (i.e. cross-reactivity immunity to *Plasmodium* infection) that can affect MA-ARDS pathogenesis.

## 4 MA-ARDS Pathogenesis

### 4.1 Sequestration of Malaria Parasites

*P. falciparum* parasites residing within the red blood cells have devised multiple strategies to survive within its host. The parasite alters the iRBCs membrane composition to express cytoadhesive ligands that interact with various adhesion receptors present on the endothelial lining. This cytoadhesive properties allow iRBCs sequestration in the endothelium bed of the organs. This allows the parasite to escape splenic clearance (Henry et al., 2020). Parasite cytoadherence to the endothelium and sequestration in deep tissues are associated to the development of severe malaria (Craig et al., 2012). Cytoadherence and sequestration of iRBCs in the endothelium causes (1) reduced blood flow in the blood capillaries/vessels and (2) activation of endothelial cells leading to inflammation and subsequent disruption of endothelium integrity (Craig et al., 2012).

In the lung sections from deceased *P. falciparum-*infected patients that developed MA-ARDS, iRBCs were found to sequester in the lung microvasculature. These *P. falciparum*-iRBCs sequestered in the vessels were predominantly the matured stages (MacPherson et al., 1985). Sequestration of *falciparum*-iRBCs to the lung microvasculature was proposed as the cause of alveolar capillaries occlusion, resulting in reduced blood volume within these capillaries (MacPherson et al., 1985; Maguire et al., 2005). As for other human *Plasmodia* species, iRBCs cytoadherence properties and sequestration ability alike *falciparum*-iRBCs are emerging. Several histological studies have reported that *P. vivax*-iRBCs were found within congested alveolar capillaries (Valecha et al., 2009; Lacerda et al., 2012). Strikingly in one of these studies, sequestered *P. vivax*-iRBC were observed in the lung sections from a patient whose parasitemia was microscopy negative. Moreover, reduced blood volume was documented in patients with *vivax*-induced MA-ARDS (Anstey et al., 2007). Together, these evidences are in support of recent reports demonstrating that parasite cytoadherence to the endothelium and deep tissue sequestration are not restricted to *falciparum* but extend to other *Plasmodia* infecting humans, such as *P. vivax* (Carvalho et al., 2010; Lopes et al., 2014) and *P. knowlesi* (Miller et al., 1971; Fatih et al., 2012; Lee et al., 2021).

It has been demonstrated that *falciparum-*iRBCs cytoadhere through several families of parasite cytoadherence ligands expressed on its surface, namely PfEMP1, STEVOR and RIFIN (Lee et al., 2019). Of the cytoadhesion interactions between the host receptor and parasite ligand, PfEMP1 with EPCR has been associated with severe malaria, particularly cerebral complications (Moxon et al., 2013; Turner et al., 2013; van der Poll, 2013). In the study by Maknitikul et al., EPCR was expressed on the lung endothelium from post-mortem tissue samples of patients with MA-ARDS, although the levels of EPCR were reported to be negatively correlated to iRBCs accumulation in the lung (Maknitikul et al., 2017). In a separate study, it was further demonstrated that PfEMP1 was able to bind to EPCR expressed on the lung endothelial cells, aside from the brain (Turner et al., 2013). In addition, another receptor that interacts with PfEMP1, namely ICAM-1, its expression was found elevated on the lung endothelium of MA-ARDS patients’ organ sections and in *in vitro* studies (Avril et al., 2016; Maknitikul et al., 2017). ICAM-1 was also identified as a receptor that *vivax*-iRBCs cytoadhere to on HLECs under flow conditions *in vitro* (Carvalho et al., 2010). Thus, demonstrating that *vivax*-iRBCs do possess the ability to sequester. This phenomenon was also recently described for *P. knowlesi*-iRBCs that CD36 was a potential binding receptor on HLECs, with enhanced binding when primed with parasite culture supernatant *in vitro* (Lee et al., 2021). Although, at present the parasite cytoadherence ligands of *vivax*-iRBCs and *knowlesi*-iRBCs are yet to be fully defined, sequestration mediated through ligands such as *vivax*-derived variant interspersed repeats (VIR) proteins and *knowlesi*-derived schizont-infected cell agglutination (SICA) proteins have been suggested (del Portillo et al., 2001; Korir and Galinski, 2006).

As sequestration is crucial for MA-ARDS pathogenesis, there is a clear need for *in vivo* studies to understand its involvement in the process of pathology development with *in vivo* conditions (such as waste clearance, circulatory flow, host immune system). Moreover, experiments with human-malaria causing *Plasmodium* iRBCs isolated from the peripheral circulation of patients with severe malaria, might be biologically different (in terms of cytoadhesive receptor specificity, affinity or avidity, and interactions) to parasite sequestered in the microvasculature. These limitations would eventually cloud the genuine associations of sequestration with the different pathologies.

With the advancement of genetic modification and *in vivo* vascular imaging techniques, infection of mouse strains with transgenic *P. berghei* parasites expressing luciferase can be used to visualize sequestration in relation to its distribution and load in various organs (Claser et al., 2014). Sequestration of luciferase-expressing *P. berghei*-iRBCs was observed in the lung of mice with MA-ARDS (Franke-Fayard et al., 2005; Sercundes et al., 2016; Vandermosten et al., 2018). In a more detailed study, Claser et al. have shown that iRBCs sequester in the perfused lungs (unbound iRBCs were removed) prior to the onset of vascular leakage, once sequestration reaches a saturable level, leakage ensues (Claser et al., 2019). Also, in the same study, the use of intensive anti-malarial treatments to remove circulatory and sequestered iRBCs prior to leakage, were sufficient to protect infected mice from lung hyperpermeability. This clearly demonstrates the importance of parasite sequestration in the lung microvasculature as a pre-requisite for lung pathology development.

Emerging studies have identified several cytoadhesion interactions between the host-derived receptors and *P. berghei*-iRBCs in the mouse lung tissue, which parallels those described with human-malaria causing *Plasmodium* iRBCs. To date, functional homolog of PfEMP1 in *P. berghei*-iRBCs has not been identified. Nonetheless, proteins such as, the Maurer’s cleft skeleton binding protein-1 (SBP-1) and histidine-rich protein (MAHRP-1), that mediate the export of parasite cytoadhesion ligands (e.g. PfEMP1) to the surface of the iRBCs, are conserved between human-malaria and rodent-malaria *Plasmodia* (De Niz et al., 2016). With the use of mutant parasites, SBP-1 and MAHRP-1 have been shown to mediate the sequestration of *P. berghei*-iRBCs in the lungs of animals, depleted of circulating erythrocytes by perfusion with a saline solution (De Niz et al., 2016; Possemiers et al., 2021). Apart from reduced parasite load in the lung, the absence of SBP-1 was shown to induce spontaneous resolution and less inflammatory responses in the lung, despite continuous presence of parasite (Possemiers et al., 2021). For *P. vivax*, members of the *vir* multigene family have been suggested to mediate cytoadherence (Carvalho et al., 2010; Bernabeu et al., 2012; Fernandez-Becerra et al., 2020; Schappo et al., 2022), bears close homology to the *P. berghei bir* multigene family (Janssen et al., 2004). In the mouse model, PbA cytoadherence to host-derived receptor, CD36, was identified to be crucial for parasite sequestration (Franke-Fayard et al., 2005; Lovegrove et al., 2008; Fonager et al., 2012; Anidi et al., 2013). This was clearly demonstrated in the study by Anidi et al. that in the absence of CD36, PbA-infected mice were shown to have reduced parasites sequestered to the alveolar capillaries and vessels, with subsequently less induction of alveolar-capillary breakdown and endothelial barrier dysfunction (Anidi et al., 2013). It has been further suggested by Fonager et al., that CD36-dependent cytoadherence to the endothelium by late stage PbA-iRBCs was mediated by the schizont membrane-associated cytoadherence (SMAC) protein, expressed in cytoplasm of the iRBC. However, the study showed that the absence of SMAC had minimal effect on sequestration in the lung during infection with the SMAC mutant PbA parasites, indicating that other parasite molecules are involved in parasite sequestration (Fonager et al., 2012). Indeed, it was suggested that sequestration of *P. berghei*-iRBCs could be mediated by several host-derived receptors such as EPCR, ICAM-1 and VCAM-1, as deficiencies in these receptors led to significant reduction in parasite load in the lung (Favre et al., 1999; Li et al., 2003; Avril et al., 2016; Dos Santos Ortolan et al., 2019). However, El-Assaad et al. have demonstrated that inhibition of ICAM-1 had no effect on the *in vitro* binding of mature stage PbA-iRBCs to lung microvascular endothelial cells (LMVECs) (El-Assaad et al., 2013). Treatment with dexamethasone in DBA/2-infected with PbA, downregulates EPCR expression in the lung and protected the mice from lung hyperpermeability (Dos Santos Ortolan et al., 2019). Nevertheless, it remains to be determined further which of these host-derived receptors are mediating parasite sequestration in the lung and subsequent pathology. Although *Plasmodium*-iRBCs from mice and humans are phenotypically different, identification of similar host-derived receptors may lead to new intervention targets.

### 4.2 Cytokines

Cytokines and chemokines are small proteins released predominantly by immune cells as part of the host immune system’s coordinated defense mechanism against foreign particles or pathogens. They are involved in the recruitment and functional activation of various immune cells. Controlled inflammatory cytokine and chemokine responses are essential for the host to eliminate the parasite growth. However, exaggerated and uncontrolled responses can lead to severe disease. Elevated levels of pro-inflammatory cytokines and chemokines in the serum, TNF-α, IL-1β, IL-6, IL-8, IL-17, IFN-γ, IP-10/CXCL10 (binds to CXCR3) and MCP-1/CCL2 (binds to CCR2) (Day et al., 1999; Armah et al., 2007; Cox-Singh et al., 2011; Kinra and Dutta, 2013; Mandala et al., 2017), and reduced levels of anti-inflammatory cytokines, TGF-β (Esamai et al., 2003; Andrade et al., 2010), have been associated with severe malaria in humans and has been described extensively for cerebral malaria. However, knowledge on these analytes from patients with MA-ARDS or bronchoalveolar lavage fluid (BALF) is limited. Cytokine profiles at the site of pathology are critical as it provide signals that dictate and perpetuate the inflammatory responses locally in the tissue and may reflect the extent of the injury. To describe cytokines that are associated with MA-ARDS pathogenesis, we will draw on findings obtained from the mouse MA-ARDS models.

Global analysis of lung tissues and BALF of infected mice with MA-ARDS revealed elevated levels of the cytokines and chemokines also reported in the serum of severe malaria in humans mainly, IL-1, IL-6, IL-8, IL-10, TNF-α, IFN-γ, MCP-1/CCL2, MIP-2/CXCL2, IP-10/CXCL10, KC/CXCL1, VEGF, PIGF (Day et al., 1999; Lovegrove et al., 2008; Epiphanio et al., 2010; Van den Steen et al., 2010; Taylor et al., 2012; Deroost et al., 2013; Pham et al., 2017; Galvao-Filho et al., 2019). To understand the role of various cytokines in MA-ARDS development, infections in the specific knockout mouse models were used as, reviewed in **Table 2**.

**Table 2:** The role of cytokines/chemokines ligand-receptors in experimental MA-ARDS.

Gene knockout (^-/-^)	Parasite used for infection	Effect on MA-ARDS development (vs wild-type). Outcome (left), Histology (Right)		Additional data	Ref
TNFα^-/-^	*P. berghei* ANKA (PbA) GFP-*luciferase*	No difference (=)	=Congested septal capillaries =Haemorrhage =Interstitial edema		(Togbe et al., 2008)
TNFR1^-/-^	PbA	=	=Edema ↓Macrophages in alveolar capillaries	(=) Parasite sequestration	(Piguet et al., 2002)
	PbNK65	=	=Histopathology scores =Edema	(=) Parasite sequestration (↓) MODCs in lung tissue	(Galvao-Filho et al., 2019)
TNFR2^-/-^	PbA	=	=Edema	(=) Parasite sequestration	(Piguet et al., 2002)
IFNγR1^-/-^	PbA Bds	↓	No edema	(↑) CD4^+^, CD8^+^ T cells, neutrophils, and macrophages in the lung tissue (↑) MCP-1/CCL2, IP-10/CXCL10 in the lung	(Belnoue et al., 2008)
IFNγ^-/-^	PbA	NR	NR	(=) CD4^+^ T cells migration markers (CXCR3, CCR5) in the lung tissue (=) CD4^+^ T cells activation/effector markers (CD62^low^, Ki67) in the lung tissue (↓) CD4^+^ T cells activation/effector markers (CD44, CD71, CD11a, CD49D, GrzB) in the lung tissue (=) CD8^+^ T cells migration markers (CXCR3, CCR5) in the lung tissue (=) CD8^+^ T cells activation/effector markers (CD62^low^, CD44, CD71, Ki67, CD49D) in the lung tissue (↑) CD8^+^ T cells activation/effector markers (CD11a) in the lung tissue (↓) CD8^+^ T cells activation/effector markers (GrzB) in the lung tissue	(Villegas-Mendez et al., 2011)
	PbA GFP-*luciferase*	↓	NR	(↓) Lung vascular leakage (↓) Parasite sequestration (↑) Total leukocytes, activated CD4^+^, activated CD8^+^, Parasite-specific Pb1^+^ CD8^+^ T cells in the lung tissue (↓) MHC I expression on lung endothelial cells	(Claser et al., 2019)
IL-12Rβ2^-/-^	PbA GFP-*luciferase*	=	=Thickened alveolar septae =Haemorrhage =Interstitial edema =Congested alveolar capillaries =Leukocytes infiltration in alveoli		(Fauconnier et al., 2012)
IL-12p35^-/-^	PbA GFP-*luciferase*	=	=Thickened alveolar septae =Haemorrhage =Interstitial edema =Congested alveolar capillaries =Leukocytes infiltration in alveoli		(Fauconnier et al., 2012)
IL-12p40^-/-^	PbA GFP-*luciferase*	=	=Thickened alveolar septae =Haemorrhage =Interstitial edema =Congested alveolar capillaries =Leukocytes infiltration in alveoli		(Fauconnier et al., 2012)
CXCL10^-/-^	PbA	↓	↓Alveolar edema ↓Leukocytes infiltration in alveoli	(↑) HO-1 levels in lung tissue, positively associated with free heme	(Liu et al., 2012)
CCR2^-/-^	PbA	↓	↓Edema	(↓) Monocytes/MDMs in the lung tissue (↑) Hz-containing cells in the lung tissue	(Lagasse et al., 2016)
	PbNK65	=	=Edema =Histopathology scores	(=) Parasite sequestration (↑) Neutrophils in the lung tissue Delayed MODCs infiltration into the lung tissue (↓) Ly6C^+^ Inflammatory monocytes in the lung tissue (=) Alveolar macrophages in the lung tissue (=) CD8^+^ T naïve/effector/central memory cells in the lung tissue (=) CD4^+^ T naïve/effector/central memory cells in the lung tissue	(Galvao-Filho et al., 2019; Pollenus et al., 2020)
CCR4^-/-^	PbNK65	↓	↓Thickened alveolar septae ↓Haemorrhage ↓Edema	(=) Parasite sequestration (↓) MODCs in the lung tissue	(Galvao-Filho et al., 2019)

TNF, Tumour necrosis factor; TNFR, Tumour necrosis factor receptor; IFN, Interferon; IFNR, Interferon receptor; IL, Interleukin; CXCL, CXC chemokine ligand; CCR, CC chemokine receptor; MODCs, Monocyte-derived dendritic cells; MDMs, Monocyte-derived macrophages; Hz, Hemozoin; (=), No difference; NR, Not reported; (↓), Decreased; (↑), Increased.

During the acute phase of blood stage infection, the febrile response is a typical host immune defense triggered by pyrogenic cytokines, IL-6 and TNF-α, produced by circulating innate cells upon recognition by its pattern recognition receptors with pathogen-associated molecular patterns (Netea et al., 2000). Moreover, in the lung, alveolar macrophages that harbor phagocytosed iRBCs and Hz may be an essential source of IL-6 and TNF-α, where the elevated levels were tightly associated with lung inflammation during the acute phase of MA-ARDS establishment (Deroost et al., 2013). In addition, pulmonary endothelial cells were found to produce TNF in DBA/2-infected with PbA (Dos Santos Ortolan et al., 2019). Although the role of IL-6 in MA-ARDS pathogenesis have yet to be reported, studies have suggested that IL-6 plays a pleiotropic role in ARDS (Butt et al., 2016). Furthermore, IL-6 can have a pro-inflammatory effect as part of the host defense mechanism in the acute phase to combat the triggering stimulant. In addition, IL-6 works in synergy with other pro-inflammatory cytokines (significantly associated with TNF-α and IL-1), causing the infiltration of various leukocyte subsets into the lung. When this response becomes excessive, it may have deleterious effects on the lung tissue resulting in injury (Tutkun et al., 2019; West, 2019; Pedersen and Ho, 2020; Ragab et al., 2020). IL-6 can have a pro-fibrotic effect in the later phase of ARDS (Le et al., 2014; Kobayashi et al., 2015). On the other hand, studies have demonstrated that TNF-α does not play a critical role in MA-ARDS pathogenesis (Piguet et al., 2002; Togbe et al., 2008; Galvao-Filho et al., 2019) (**Table 2**). Nonetheless, more studies are needed to understand the role of IL-6 and TNF-α during the establishment of MA-ARDS, and its association with circulatory levels in the serum to evaluate the possibilities as early biomarkers to determine the onset, severity, and outcome of lung injury.

IFN-γ is an essential cytokine induced and has been found to inhibit *Plasmodium* blood stages. IFN-γ is produced by various immune cell subsets from the innate and adaptive immunity arms at different phases of the blood stage infection (Gun et al., 2014). At the early phase of the blood stage infection, NK cells, NKT cells, γδ T cells, and T cells were the main producers, followed by a shift of the role to the T cells (CD4^+^ and CD8^+^) at the later phase of infection (King and Lamb, 2015). High levels of IFN-γ at the early phase of the blood stage infection have been largely correlated to protection from severe malaria and control of systemic parasite growth, but may increase the risk of developing severe malaria in the later phase of infection (King and Lamb, 2015). IFN-γ was shown to play a pathogenic role in MA-ARDS development, as the infection of IFN-γ signaling-deficient mouse models were protected from lung injury despite the increased transmigration and infiltration of leukocytes and activated effector T cells in the lung tissue (Belnoue et al., 2008; Villegas-Mendez et al., 2011; Claser et al., 2019; Galvao-Filho et al., 2019) (**Table 2**). Besides, IFN-γ was shown to be required for the maturation of TNF-a/iNOS-producing monocyte-derived dendritic cells that could augment CD8^+^T cells cytotoxicity to cause lung injury in PbNK65-infected mice (Galvao-Filho et al., 2019). Expression of other pro-inflammatory cytokines were found to be inducible by the canonical IFN-γ cytokine during lung inflammation, such as IP-10/CXCL10 (which binds to its cognate CXCR3 receptor) (Liu et al., 2012), may work in synergy with IFN-γ to promote leukocytes trafficking into the lung to cause the pathology.

### 4.3 Endothelial Cells

The pulmonary vasculature is formed by a layer of endothelial cells, serving as a semipermeable barrier that separates the pulmonary circulation from the air. It regulates lung homeostasis through intercellular junctions, allowing the paracellular transport of macromolecules and nutrients among the cells (Mehta and Malik, 2006). Disruption of this endothelial barrier causes fluid leakage from the blood vessels into the alveolar lumen, promoting pulmonary edema, recruitment of inflammatory mediators, and accumulation of leukocytes and platelets (Ware, 2006; Millar et al., 2016). During *Plasmodium* infection, the lung vasculature was suggested to be a niche for the parasite to initiate the blood stage in the host. The merosomes originating from the liver schizonts are likely disrupted within the pulmonary capillary, releasing merozoites that will infect the red blood cells. This early interaction may trigger an inflammatory response; however, it would be limited due to the low number of parasites present in the organ (Souza et al., 2013; Aitken et al., 2014).

#### 4.3.1 Endothelial Cells: Adhesion Molecules and Cytokines

Endothelial cells can be activated by vascular endothelial growth factor (VEGF), also known as VEGF-A (Kim et al., 2011), TNF-α, and by mediators produced by the *Plasmodium* parasite during infection (Gillrie et al., 2007). The activated endothelial cells release cytokines that upregulate the expression of adhesion molecules (P-, L- and E-selectin) (Combes et al., 2004; Krishnamurthy et al., 2015). Chemokines such as CCL2, CXCL4, CCL5 (Van den Steen et al., 2010), promote the adhesion of iRBCs, macrophages (Grau et al., 1987), platelets (Wassmer et al., 2004), neutrophils (Souza et al., 2013) and plasma microparticles (Faille et al., 2009) to the endothelium. IFN-γ, TNF-α and IL-1, produced by NK and mononuclear cells (alveolar and interstitial macrophages), are the most prevalent cytokines involved in leukocytes chemotaxis and for ICAM-1/CD54 and VCAM-1/CD106 expression on the endothelial cells (Smith et al., 2019). IFN-γ activate and enhance the antigen-cross presentation capability of LMVECs, through signaling the upregulation of MHC-I, thus driving the cross-presentation of immunogenic parasite epitope by LECs to cytotoxic CD8^+^ T cells. The consequence of this process is lung endothelium-epithelium hyperpermeability (Claser et al., 2019). Additionally, some studies have demonstrated that IFN-γ and TNF upregulate the expression of adhesion proteins, such as ICAM-1 and VCAM-1, promoting the binding of iRBCs on LMVECs and trafficking of leukocytes into the lung tissue of infected humans oand mice (Ockenhouse et al., 1992; Zielinska et al., 2017).

Activated endothelial cells also release Weibel-Palade bodies (WPBs), which are storage granules containing Von Willebrand factor (VWF) and angiopoietin-2 (Ang-2) (Dole et al., 2005). During endothelial injury, VWF and Ang-2 are released, and have been associated with mortality in patients with MA-ARDS (Yeo et al., 2008; Park et al., 2012; Graham et al., 2016). In one study, *vwf*-deficient mice infected with PbNK65, had reduced alveolar leakage, but surprisingly exacerbated mortality (Kraisin et al., 2019). Endothelium remodeling by Ang-2 was shown to be a physiological condition-dependent mechanism (de Jong et al., 2016). Moreover, Ang-2 and Ang2^+^-expressing leukocytes were found increased in the alveolar space of lung tissue sections from humans that died of MA-ARDS (Pham et al., 2019).

#### 4.3.2 Vascular Permeability Alterations

Leakage of solutes, cells, both small and larger molecules from the microvasculature into the tissue can occur in normal physiological condition and greatly increased in pathological state. This extravasation can happen by transcellular or paracellular mechanisms, or *via* destabilization of endothelial junctions. In pathological state, increased vascular permeability, a hallmark of ARDS, results in edema (Claesson-Welsh, 2015; Wettschureck et al., 2019).

Many inflammatory factors, such as VEGF, histamine, bradykinin, TNF-α, and IFN-γ, promotes cytoskeleton contraction and alteration on the endothelial junctions by opening the paracellular junctions, which contributes to the inflammatory process (Claesson-Welsh, 2015; Ji et al., 2021). The Rho family of GTPases, whose principal members are Cdc42, Rac, and RhoA, are dynamic regulators of the cytoskeleton, acting as molecular switches that controls cellular processes by hydrolysis of GTP, contributing to different cellular functions (Norbert Voelkel, 2009; Liu et al., 2014). RhoA and RhoB interacts with their effector Rho kinase (ROCK). RhoB regulates endothelial barrier function during inflammation by activating NF-kβ (Vincent et al., 2004). On the other hand, activation of RhoA and its effector ROCK induced by the adhesion of *P. falciparum*-iRBCs to human LMVECs, inhibits myosin phosphatase and increases myosin light chain (MLC) phosphorylation. This phosphorylation induces actomyosin polymerization, contraction, and weakening of inter-endothelial junctions, causing the increase in vascular permeability (McKenzie and Ridley, 2007; Norbert Voelkel, 2009; Beckers et al., 2010; Spindler et al., 2010; Marcos-Ramiro et al., 2014). However, excessive or abnormal activation of RhoA and its effector ROCK, by the factors such as TNF-α, oxidative stress, thrombin, growth factors, and other agents, is associated with decreased endothelial barrier function (Dejana et al., 1999; Zhao et al., 2014; Hartmann et al., 2015). In addition, TNF-α disorganizes the inter-endothelial junctions and causes disarrangement in the cytoskeleton of endothelial cells, thus increasing vascular permeability (Taoufiq et al., 2008; Zang-Edou et al., 2010; Taoufiq et al., 2011; Liu et al., 2014). Moreover, it was demonstrated that human LMVECs stimulated with *P. falciparum* extract (parasite sonicate), had increased vascular endothelial permeability due to morphological changes in adherent and occlusion junctions (Gillrie et al., 2007).

VEGF is a potent pro-angiogenic cytokine that remodels the endothelium permeability,surface adhesion molecules and mediates the chemotaxis of leukocytes (such as monocytes/macrophages) during inflammation (Heil et al., 2000; Reinders et al., 2003). It was also shown to affect endothelial barrier dysfunction by activating the Rho-GTPase signaling cascade (Gallego-Delgado et al., 2016). Excessive production of VEGF during MA-ARDS was suggested to induce lung capillary hyperpermeability (Ware, 2006; Pham et al., 2017; Pham et al., 2019). In addition, an increase of VEGF-A and PlGF in the lungs, was dependent on effector CD8^+^T cells to cause alveolar edema (Pham et al., 2017). Using DBA/2- infected with PbA, Epiphanio et al. demonstrated that high levels of circulating VEGF correlated with MA-ARDS. Intravenous infection with sFLT1 (soluble form of the VEGF receptor)-expressing adenovirus, protected the PbA-infected DBA/2 micefrom MA-ARDS and this protection was correlated with decreased levels of VEGF in the circulation (Epiphanio et al., 2010). However, the treatment with a neutralizing monoclonal antibody against VEGF receptor-2 (VEGFR-2), did not prevent MA-ARDS in PbNK65-infected C57BL/6 mice (Pham et al., 2017). Therefore, the role of VEGF in MA-ARDS is still being debated, whether they play an effector role in causing endothelium permeability or released as a result of lung injury.

Primary LMVECs (PLMVECs) from uninfected DBA/2 mice in contact with PbA-iRBCs or its lysate for one hour, induced the production of pro-inflammatory cytokine, TNF-α, cytoskeleton contraction and promoted pulmonary vascular hyperpermeability (Pereira et al., 2016; Dos Santos Ortolan et al., 2019). Furthermore, the adhesion of iRBCs to ICAM-1, leads to alterations in the conformation of actin microfilaments (forming a cup-like structure), which was proposed to be the mechanism associated with the disruption of the blood-brain barrier in the human brain endothelial cell lines (Jambou et al., 2010). On the other hand, it was demonstrated that LMVECs obtained from uninfected DBA/2 mice previously treated with hemin, induces heme oxygenase-1 (HO-1) and an anti-inflammatory effect, which decreases cytoskeleton contraction. This consequently reduced the opening of the inter-endothelial junctions (Pereira et al., 2016).

**Figure 1:**
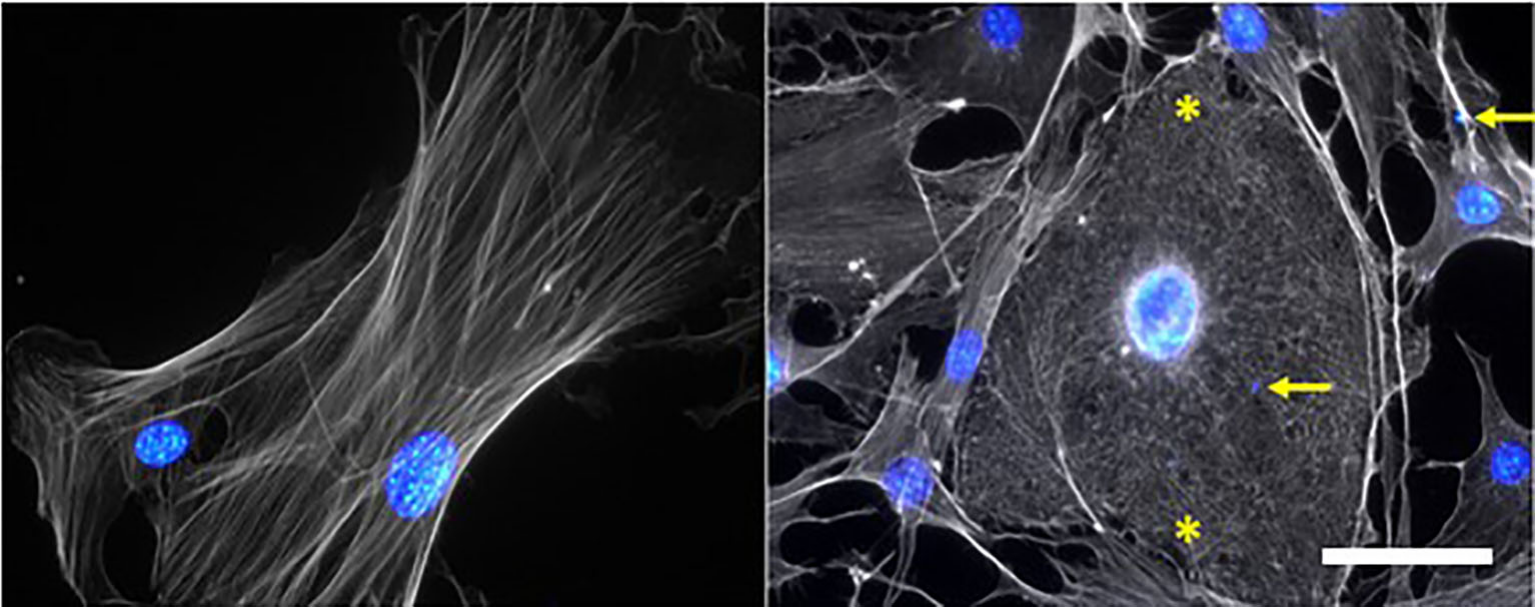
*P. berghei* ANKA-infected red blood cells (PbA-iRBCs) generate morphological alterations in the cytoskeleton of primary pulmonary endothelial cells (PMLECs) of DBA/2 mice. Non-stimulated (NS) cell show elongated actin microfilaments, while adhered PbA-iRBCs cause shortening and entanglement of these filaments, indicated by asterisks. Actin and cell nuclei were stained with Texas Red Phalloidin], Hoechst [1:1000], respectively. Yellow arrows point to PbA-iRBCs nuclei; Scale bar: 50 μm.

Studies in murine models and patients have shown the involvement of the epithelial and endothelial cell death in the pathogenesis of ARDS (Bachofen and Weibel, 1977; Fujita et al., 1998). Apoptosis involving the Fas/FasL pathways and activation of caspases have been suggested to mediate pathogenesis in the lung of *P. falciparum*-infected patients with pulmonary edema (Taoufiq et al., 2008; Taoufiq et al., 2011; Punsawad et al., 2015). PLMVECs in contact with PbA-iRBCs for 24 hours showed increased endothelial permeability induced by the activation of caspases mechanism, leading to apoptosis of these cells (Sercundes et al., 2022). The contact between PbA-iRBCs and PLMVECs from DBA/2 mice induce alterations in the conformation of actin microfilaments, with a short network and cross-linked filaments, compared to actin from non-stimulated cells with long, parallel fibers arranged longitudinally (**Figure 1**). The sepsis model demonstrated that cell-free hemoglobin increased lung apoptosis, contributing to endothelial injury and increased vascular permeability (Meegan et al., 2020). Kingston and collaborators showed that cell-free hemoglobin levels were higher in patients with severe malaria than uncomplicated malaria or healthy controls. In addition, cell-free hemoglobin diminishes peripheral perfusion (related to mortality) by NO scavenging (Kingston et al., 2020). Using PbA-infected C57BL/6 mice, Anidi et al. demonstrated that the increase in pulmonary endothelial permeability after cytoadhesion of iRBCs, was mediated by CD36 and Fyn kinase (Anidi et al., 2013).

Treating and preventing ARDS is essential to protect and restore pulmonary endothelial barriers, and to combat endothelial dysfunction. Some molecules such as steroidal anti-inflammatory, RhoA inhibitors, histamine receptor blockades, sphingosine 1-phosphate, angiopoietins, PKC inhibitors, anti-VEGF, and others, have displayed the ability to improve endothelial barrier properties (Ma et al., 2019). Annexin A1, for instance, preserves junction integrity and consequently decreases vascular permeability (Ma et al., 2019; He et al., 2021). Ac2-26, an annexin A2 peptide, was demonstrated to reduce systemic inflammation through decreasing leukocyte migration to the lung, induction of anti-inflammatory IL-10 production (Guido et al., 2013), reducing oxidative stress responses and protecting from ARDS in the LPS model (Ju et al., 2021). According to He and colleagues, this peptide promotes phosphorylation of PI3K and Akt, and inhibits p-NF-κB (p65) expression, reducing pulmonary inflammation and injury (He et al., 2021). Rho-kinase inhibition by fasudil (a potent and selective Rho kinase inhibitor) can be a pertinent approach to reduce the effects of severe malaria, including ARDS patients (Taoufiq et al., 2008). Caspase inhibition by ZVAD-fmvk prevented the increase of pulmonary vascular permeability *in vivo* (PbA-infected DBA/2 mice), and *in vitro* (PbA-iRBCs in PLMVECs for one hour). In addition, PbA*-*infected DBA/2 mice treated with hemin were protected from MA-ARDS development, especially concerning the alveolar-capillary barrier (Pereira et al., 2016). As much as the endothelium plays a fundamental role in the development of MA-ARDS, drugs that modulate vascular permeability or reduce the inflammatory processes in the lung tissue, are essential targets. However, most of the current studies are done *in vitro* or are still in pre-clinical trials, and there is still a long way before it can be prescribed to malaria patients, especially with lung injuries.

### 4.4 Leukocytes

Malaria infection incites the inflammation process, triggering intense leukocyte infiltration into the lung parenchyma (Deroost et al., 2013; Aitken et al., 2014). The expression of CAMs on activated endothelial cells and activation of P- and E-selectin, induce the expression of β2-integrins, leading to subsequent leukocyte arrest in the vasculature (Krishnamurthy et al., 2015). The accumulation of these leukocytes in the capillary and alveolar space, and the interaction between leukocyte and endothelium, observed in both humans and animals, were shown to contribute to MA-ARDS pathogenesis (Taylor et al., 2012; Souza et al., 2013). Among the infiltrated leukocytes documented, monocytes, macrophages, lymphocytes, and neutrophils were found in the lung of patients and in the mouse models (reviewed in (Van den Steen et al., 2013).

#### 4.4.1 Monocytes/Macrophages

Upon iRBCs sequestration in the pulmonary vasculature, monocytes/macrophages were found in the lung tissue of *P. falciparum*-infected patients with MA-ARDS (Mohan et al., 2008) and PbA-infected mice. These cells mediate the clearance of iRBCs in a CD36-dependent manner. In addition, CCR2^+^CD11b^+^Ly6C^hi^ monocytes from the circulation were found to accumulate in the lungs as monocyte-derived macrophages (MDM) (Lagasse et al., 2016). Furthermore, it was demonstrated that infection of CD36 bone marrow chimeric mice resulted in the phagocytic clearance by monocytes, leading to exaggerated lung pathology (Lagasse et al., 2016). The increase in MDM in the lung on 7 days post-infection (dpi) of PbA-infected C57BL/6 mice was further corroborated by Claser et al. (2019).

Monocytes/macrophages when activated by parasites components, including Hz, secrete pro-inflammatory cytokines that are likely to contribute to MA-ARDS pathogenesis (Mohan et al., 2008). Monocytes and macrophages, with or without ingested Hz, were found sequestered in the lung tissue in both humans (Duarte et al., 1985; Valecha et al., 2009; Lacerda et al., 2012) and mice (Van den Steen et al., 2010; Hee et al., 2011). Hz is shown to be involved in the pathogenesis of MA-ARDS through inflammasome activation in the monocytes/macrophages, thus releasing IL-1β and IL-18 (Dolinay et al., 2012), and causes M1 polarization of activated macrophages that produces pro-inflammatory cytokines (TNF-α, IL-6) (Mills et al., 2000). Hz-containing iRBC were frequently observed in congested small vessels in the lungs of PbA-and PbNK65-infected mice (Deroost et al., 2013). Deroost et al. demonstrated that injection of PfHz into *Plasmodium*-free mice (absence of *Plasmodium* infection), induced pulmonary expression of chemokines (IP-10/CXCL10, MCP-1/CCL2, KC), cytokines (IL-1ß, IL-6, IL-10, TNF, TGF-β), and inflammatory mediators (iNOS, NOX2, Hmox1, ICAM-1), that are associated with alveolar edema (Deroost et al., 2013). During lung injury, recruitment of monocytes/macrophages expressing CCR2^+^ (Hartl et al., 2005), was shown to be MCP-1/CCL2-dependent. In CCR2-deficient mice, PbA infection induces a marginal increase in the lung edema of PbA-infected CCR2-deficient mice (Lagasse et al., 2016), and with PbNK65 infection no improvement in survival was reported (Galvao-Filho et al., 2019), compared to wild-type (WT) mice. Moreover, in this same study done by Lagasse et al., lung pathology still occurred despite hampered MDM recruitment into the lungs, thus excluding MDM as an important mediator of MA-ARDS (Lagasse et al., 2016). Using antimalarial treatment, it was demonstrated that despite monocytes being retained in the bone marrow of CCR2-deficient mice, there was no effect on the development and resolution of MA-ARDS. On the other hand, CCR2 is needed to re-establish the homeostasis of pulmonary leukocytes during recovery (Pollenus et al., 2020).

In the lung, there are two main types of macrophages (1) alveolar macrophages (AMs), located at the air-tissue interface and are the predominant cells present in the alveolus; (2) interstitial macrophages (IMs), located in the space between the alveolar epithelium and vascular endothelium (Schneberger et al., 2011). AMs, also known as tissue-resident macrophages, are the second line of defense against pathogens. Upon infection, these cells undergo a remodelling process, expressing MHC-II on their surface (Guillon et al., 2020). During the earlier stages of MA-ARDS, there are little evidences of AMs activation and proliferation in response to parasite sequestration in the pulmonary vasculature (Lagasse et al., 2016). Recently, Goggi et al. observed that starting on 6 dpi and peaking on 7 dpi, there is an increase in inflammatory macrophages and AMs expressing MHC-II in the lung of PbA-infected C57BL/6 mice. Moreover, using PET imaging, the group observed that the increase in both populations correlated with the retention of [^18^F]FEPPA uptake, a PET radioligand developed to target the 18-kDa translocator protein (TSPO) overexpressed in activated macrophages (Goggi et al., 2021). Although several studies have suggested the role of monocytes and macrophages in the pathogenesis of MA-ARDS, more studies are required to uncover whether these immune cells subset could augment the trafficking of other subsets to the lung to induce injury.

#### 4.4.2 Neutrophils

Neutrophils are important for the pathophysiology of various lung injuries (Abraham, 2003). Migration and adhesion to the lung alveoli by neutrophils are strategic to begin the inflammatory process (Reid and Donnelly, 1996). In murine models of ARDS, neutrophils can control endothelial barrier function either by adhering on the endothelial cells or by secretion of their compounds (Ma et al., 2019). Neutrophil adhesion to endothelial cells increases Src phosphorylation of caveolin 1 (through ICAM-1 signaling), forming caveolae’s and augmenting transcellular permeability in the lung, thus contributing to edema formation (Hu et al., 2008). Neutrophils discharge (from extracellular vesicles or granular contents) or neutrophils extracellular traps (NETs), can also increase paracellular permeability, promoting gap formation by cytoskeleton contraction, junction disruption, focal adhesion reorganization, and glycocalyx degradation. This secretion-dependent mechanism occurs after the release of ROS, metalloproteinases, elastase, S100A8, S100A9, MPO, cathepsin G, and inflammatory mediators (Ma et al., 2019).

Neutrophils (with or without) phagocytosed Hz are frequently observed in alveolar and interstitial space in the lung with *Plasmodium* infection, associated with iRBCs, monocytes and lymphocytes (MacPherson et al., 1985; Taylor et al., 2012). Although the role of neutrophils in malaria has long been neglected, studies have recently reported both protective and pathogenic functions. Ampawong et al. have identified elastase and neutrophils in the lung of Southeast Asian patients with severe *P. falciparum*, with or without pulmonary edema (Ampawong et al., 2015). On the other hand, neutrophils (identified by the CD15 marker) were detected in lung sections from deceased Brazilian patients infected by *P. vivax* (Lacerda et al., 2012). In murine models, several studies have reported elevated neutrophils in the BALF of PbA-infected DBA/2 mice on 7 dpi (Sercundes et al., 2016), or PbA-infected C57BL/6 mice on 7-8 dpi (Van den Steen et al., 2010), and PbNK65-infected C57BL/6 mice on 10 dpi (Van den Steen et al., 2010). However, findings by Claser et al. reported no significant difference in neutrophil numbers in the lung tissue of PbA-infected C57BL/6 mice on 7dpi (Claser et al., 2019). In PbA-infected DBA/2 mice, neutrophils was shown to be essential to the pathogenesis of ARDS-developing mice releasing ROS, myeloperoxidase (MPO), as well as NETs (Sercundes et al., 2016), an important mechanism to capture and kill parasite outside the cell. In this same study, the authors demonstrated that the increase in mouse survival and decreased MA-ARDS development was possible when neutrophils were depleted with AMD3100 (CXCR4 antagonist), NET formation was blocked with Sivelestat (inhibitor of neutrophil elastase) and NETs were destroyed with Pulmozyme (human recombinant DNase) (Sercundes et al., 2016). Upon neutrophils adhesion to the endothelial cells, ROS and proteases are released, leading to the necrosis of endothelial cells and, thus increasing pulmonary vascular permeability as shown in other experimental system (Ma et al., 2019). In addition, extracellular heme-induced NETs formation, followed by its disintegration (by plasma DNase I), activates the endothelial cells and, consequently enables iRBCs sequestration, contributing to disease severity (Knackstedt et al., 2019).

#### 4.4.3 T Cells

During MA-ARDS, lymphocytes (T cells) are recruited in and around the pulmonary vasculature of patients and mouse models. These cells are also capable of secreting cytokines (TNF-α, IFN-γ, IL-1) that enhance the local inflammatory response leading to endothelial dysfunctions (Chang et al., 2001). Among CD3^+^ lymphocytes, CD8^+^T cells were demonstrated to be the main effector cells of MA-ARDS in PbA-infected C57BL/6 mice (Claser et al., 2019). Furthermore, it was shown that during infection, lung endothelial cellsactivated by IFN-γ produced systemically, acquires the capacity to capture, process and cross-present the PbA parasite antigen to parasite-specific CD8^+^T cells (Claser et al., 2019). Depletion of CD8^+^T cells with monoclonal antibody significantly reduced pulmonary vascular leakage, alveolar edema, and leukocyte infiltration in infected mice (Chang et al., 2001; Van den Steen et al., 2010; Pham et al., 2017; Claser et al., 2019). Moreover, in the absence of CD8^+^ T cells, parasite density was shown to be decreased in the lung of PbA-infected C57BL/6 mice on 7 dpi. In addition, tight junction protein *zonula occludens*-1 (ZO-1) expression on the lung epithelium was also significantly maintained (Claser et al., 2019). Vascular permeability factors, such as VEGF-A and placental growth factor (PIGF), were found reduced in the lung of PbNK65-infected C57BL/6 mice depleted of CD8^+^T cells (Pham et al., 2017). Treatment with dexamethasone decreased CD8^+^T cells and macrophages in the lung of PbNK65-infected C57BL/6 mice, conferring sufficient protection from MA-ARDS (Van den Steen et al., 2010). Galvão-Filho et al. demonstrated that IFN-γ produced by CD8^+^T cells was required for inducing the differentiation of TNF-a/iNOS-producing monocyte-derived dendritic cells in the lung, and for the development of MA-ARDS in PbNK65-infected C57BL/6 mice (Galvao-Filho et al., 2019). This finding suggested that infiltrating monocytes and dendritic cells could augment CD8^+^T cells migration and cytotoxicity leading to lung injury (Yeo et al., 2008).

Besides the conventional T cells that express CD4^+^ or CD8^+^, there is a minor population of innate lymphocytes, known as gamma delta T cells (γδ T cells), that are also found in the lung during MA-ARDS (Wei et al., 2021). These cells are known to respond to antigen without presentation (Lawand et al., 2017). Using γδ T cells-deficient mice infected with *P. yoelii*, lung injury still ensued despite the decrease in absolute number of total CD3^+^ cells including, CD4^+^ and CD8^+^T cells. On the other hand, the percentage of IFN-γ-expressing CD3^+^ and CD8^+^ cells were higher in infected- γδ T deficient mice compared to WT mice (Wei et al., 2021). Even though γδ T cells contribute to T cell immune response in the lung of *Plasmodium*-infected mice, further studies are needed to understand their role in MA-ARDS development.

### 4.5 Platelets

Platelets are anucleated cells traditionally known for their role in thrombosis and hemostasis. In the last few decades, these cells were shown to play a dual role in the pathogenesis of *Plasmodium* infection, by preventing the exponential growth of parasitemia in the early stage, and promoting enhanced immune responses in the later stage (Srivastava, 2014). Platelets were also associated with the pathogenesis and progression of MA-ARDS, because upon its activation, they release various inflammatory mediators (von Willebrand factor, PF4/CXCL4, RANTES/CCL5) that activate neutrophils, monocytes, macrophages, leukocytes, and endothelial cells, leading to pulmonary endothelial damage (Vieira-de-Abreu et al., 2012; Srivastava, 2014; de Azevedo-Quintanilha et al., 2020).

During *Plasmodium* infection, platelets can bind to non-infected and iRBCs and form agglutinates. The interaction between the platelet and iRBCs is mediated through CD31/PECAM-1 and CD36 expressed on platelets with PfEMP-1 expressed on iRBCs (Pain et al., 2001). Platelets also can act as an adhesive bridge between *P. falciparum*-iRBCs and activated endothelial cells (Wassmer et al., 2004). Studies done either *in vitro* using human platelets or in *Plasmodium*-infected mice depleted of platelets demonstrated that platelets are responsible for the killing of iRBCs, a mechanism involving platelet factor 4 (PF4/CXCL4) (McMorran et al., 2009; McMorran et al., 2012). PF4 is a chemokine released from intracellular granules upon platelet activation and has been found highly elevated in malaria patients and mouse models (Srivastava et al., 2008). PF4 was shown to engage the Duffy antigen receptor expressed on iRBCs, inducing the disruption of parasite digestive vacuole without lysing the iRBCs (McMorran et al., 2012), and to bind to CXCR3^+^-expressing cells (monocytes, macrophages, leukocytes), thus being responsible for their activation and attachment to the endothelium (Srivastava, 2014).

Besides iRBCs, platelets can interact with neutrophils and leukocytes. When the receptor P2Y1 is stimulated on platelets, RhoA pathway is activated, resulting in platelet-leukocyte aggregation, followed by migration to the lung and finally binding to the pulmonary endothelium (Amison et al., 2015). The leukocyte-endothelium interaction is further enhanced by microparticles released by the platelets, which stimulates the neutrophils to upregulate their αM integrin expression, allowing its adhesion to LMVECs *via* ICAM-1 (Xie et al., 2015). The interaction between platelets-neutrophils and platelets-leukocytes, results in the activation of leukocytes and neutrophils, contributing to MA-ARDS pathogenesis (Srivastava, 2014). Platelets are also known to have the capacity to activate the classical and alternative pathway of the complement system. As a result of this activation, there is an increase in the inflammatory mediators, such as C3a and C5a, resulting in neutrophil activation thus leading to pulmonary capillary and alveoli damage (Peerschke et al., 2010; Bosmann and Ward, 2012). Using PbA-infected mice, Piguet et al. observed that when the platelet activation was hindered, the leukocyte adhesion to the lung vasculature was decreased (Piguet et al., 2000). Moreover, they also demonstrated that during PbA-infection, the sequestration of platelets in the lung is dependent on urokinase-type plasminogen activator (uPA). In uPA receptor deficient mice infected with PbA, had a prolonged survival with no difference in pulmonary permeability compared to WT mice (Piguet et al., 2000). Recently, another group demonstrated that PbA-infected Nbeal2-deficient mice, which have less platelets, were protected from pulmonary vascular permeability compared to control mice (Darling et al., 2020). Platelet-activating factor (PAF), another mediator of inflammation, is involved in the recruitment and activation of leukocytes, release of cytokines and chemokines, and vascular permeability factor (Chao and Olson, 1993). The role of PAF in the pathogenesis of pulmonary damage was demonstrated in PbA-infected platelet-activating factor receptor (PAFR)-deficient mice. These mice had decreased lung damage characterized by lesser infiltration of neutrophils, macrophages and CD8^+^T cells in the alveolar space (Lacerda-Queiroz et al., 2013).

Platelets are involved in thrombo-inflammation during malaria infection. Thrombocytopenia is a complication observed in infected patients (predominantly with *vivax* or *falciparum* infection) (Lacerda et al., 2011; Bakhubaira, 2013) and mouse models (de Azevedo-Quintanilha et al., 2020), and has been correlated with increase parasite density and disease severity (Gerardin et al., 2002; Ladhani et al., 2002). The mechanism of platelet clearance observed during thrombocytopenia has been widely studied and is still being debated. Some studies have suggested that it is associated with platelet activation (Lacerda et al., 2011; Sharron et al., 2012), followed by platelet adhesion to the endothelium, resulting in endothelial cell activation causing the release of VWF, and hetero-aggregates of platelet and leukocytes (de Azevedo-Quintanilha et al., 2020). When the endothelium is activated, coagulation related proteins are released, such as thrombomodulin and its ligand, thrombin, leading to platelet consumption (Thachil, 2017). On the other hand, Mast et al. demonstrated that thrombocytopenia could happen in the absence of platelet activation or disseminated intravascular coagulation (de Mast et al., 2010). Treatment with heme oxyganese-1 inducer cobalt protoporphyrin IX (CoPPIX) reduced thrombocytopenia and decreased inflammatory infiltrate in the lung parenchyma in PbNK65-infected mice (de Azevedo-Quintanilha et al., 2020). Although a few studies have been conducted to understand the role of platelets in the MA-ARDS pathogenesis, great caution must be taken in platelet-targeted therapeutic treatment. Anti-platelet treatment is highly discouraged in malaria patients with thrombocytopenia. Instead, it was proposed that treatment should target the platelet-leukocytes and platelet-neutrophils interactions (Srivastava, 2014).

## 5 Conclusion

In this review, we draw attention to the importance of using animal models to understand how inflammatory responses and the endothelial activation during MA-ARDS can orchestrate endothelial dysfunction, leading to increase vascular permeability and disease development. Several mouse MA-ARDS models have been developed and infection of knockout mice have been used to better understand the mechanisms involved in the pathogenesis of MA-ARDS. In the last few years, sophisticated imaging procedures such as SPECT/CT, MRI and PET scans conducted in mice (particularly in the conscious state), have helped to precisely assess early onset of MA-ARDS and track the disease progression. Gaining knowledge is imperative to provide the mechanistic basis and targets to develop adjunct therapies, which are currently unavailable. Although much has been done to uncover the immune responses and mechanisms underlying MA-ARDS pathogenesis, more remains to be done to understand how these responses can be modulated, potentially translating into viable treatments. It is clear that animal models of MA-ARDS are pivotal for the development of methods and tools for early diagnosis to assess and predict the severity of disease and guide the development of therapeutic approaches.

## Author Contributions

CC conceptualized the review. JD, SN, SE, CC, and LR wrote the draft. All authors contributed to the article and approved the submitted version.

## Funding

CC was financially supported by 2018/24470-0 grant from the São Paulo Research Foundation (FAPESP). JD was supported by Coordination for the Improvement of higher Education Personnel (Coordenação de Aperfeiçoamento de Pessoal de Nível Superior: CAPES, Brazil) fellowship. SE was supported by 2020/03163-1 from the São Paulo Research Foundation (FAPESP) and 304033/2021-9 from National Council for Scientific and Technological Development (Conselho Nacional de Desenvolvimento Científico e Tecnológico: CNPq, Brazil). LR was supported by Agency for Science, Technology and Research (A*STAR) to a core grant to A*STAR ID labs and a Starting University grant from the Lee Kong Chian School of Medicine, Nanyang Technology University. SN was supported by a postgraduate scholarship from the Yong Loo Lin School of Medicine, National University of Singapore.

## Conflict of Interest

The authors declare that the research was conducted in the absence of any commercial or financial relationships that could be construed as a potential conflict of interest.

## Publisher’s Note

All claims expressed in this article are solely those of the authors and do not necessarily represent those of their affiliated organizations, or those of the publisher, the editors and the reviewers. Any product that may be evaluated in this article, or claim that may be made by its manufacturer, is not guaranteed or endorsed by the publisher.

## References

[B1] Abraham E. (2003). Neutrophils and Acute Lung Injury. Crit. Care Med. 31 (4 Suppl), S195–S199. doi: 10.1097/01.CCM.0000057843.47705.E8 12682440

[B2] Aitken E. H. Negri E. M. Barboza R. Lima M. R. Alvarez J. M. Marinho C. R.. (2014). Ultrastructure of the Lung in a Murine Model of Malaria-Associated Acute Lung Injury/Acute Respiratory Distress Syndrome. Malar. J. 13, 230. doi: 10.1186/1475-2875-13-230 24927627 PMC4062769

[B3] Amison R. T. Momi S. Morris A. Manni G. Keir S. Gresele P.. (2015). RhoA Signaling Through Platelet P2Y(1) Receptor Controls Leukocyte Recruitment in Allergic Mice. J. Allergy Clin. Immunol. 135 (2), 528–538. doi: 10.1016/j.jaci.2014.09.032 25445826

[B4] Ampawong S. Chaisri U. Viriyavejakul P. Prapansilp P. Grau G. E. Turner G. D.. (2015). A Potential Role for Interleukin-33 and Gamma-Epithelium Sodium Channel in the Pathogenesis of Human Malaria Associated Lung Injury. Malar. J. 14, 389. doi: 10.1186/s12936-015-0922-x 26437894 PMC4595310

[B5] Andrade B. B. Reis-Filho A. Souza-Neto S. M. Clarencio J. Camargo L. M. Barral A.. (2010). Severe Plasmodium Vivax Malaria Exhibits Marked Inflammatory Imbalance. Malar. J. 9, 13. doi: 10.1186/1475-2875-9-13 20070895 PMC2837053

[B6] Anidi I. U. Servinsky L. E. Rentsendorj O. Stephens R. S. Scott A. L. Pearse D. B. (2013). CD36 and Fyn Kinase Mediate Malaria-Induced Lung Endothelial Barrier Dysfunction in Mice Infected With Plasmodium Berghei. PloS One 8 (8), e71010. doi: 10.1371/journal.pone.0071010 23967147 PMC3744507

[B7] Anstey N. M. Handojo T. Pain M. C. Kenangalem E. Tjitra E. Price R. N.. (2007). Lung Injury in Vivax Malaria: Pathophysiological Evidence for Pulmonary Vascular Sequestration and Posttreatment Alveolar-Capillary Inflammation. J. Infect. Dis. 195 (4), 589–596. doi: 10.1086/510756 17230420 PMC2532499

[B8] Anstey N. M. Jacups S. P. Cain T. Pearson T. Ziesing P. J. Fisher D. A.. (2002). Pulmonary Manifestations of Uncomplicated Falciparum and Vivax Malaria: Cough, Small Airways Obstruction, Impaired Gas Transfer, and Increased Pulmonary Phagocytic Activity. J. Infect. Dis. 185 (9), 1326–1334. doi: 10.1086/339885 12001051

[B9] Armah H. B. Wilson N. O. Sarfo B. Y. Powell M. D. Bond V. C. Anderson W.. (2007). Cerebrospinal Fluid and Serum Biomarkers of Cerebral Malaria Mortality in Ghanaian Children. Malar. J. 6, 147. doi: 10.1186/1475-2875-6-147 17997848 PMC2186349

[B10] Avril M. Bernabeu M. Benjamin M. Brazier A. J. Smith J. D. (2016). Interaction Between Endothelial Protein C Receptor and Intercellular Adhesion Molecule 1 to Mediate Binding of Plasmodium Falciparum-Infected Erythrocytes to Endothelial Cells. mBio 7 (4), 1–10. doi: 10.1128/mBio.00615-16 PMC4958245 27406562

[B11] Bachofen M. Weibel E. R. (1977). Alterations of the Gas Exchange Apparatus in Adult Respiratory Insufficiency Associated With Septicemia. Am. Rev. Respir. Dis. 116 (4), 589–615. doi: 10.1164/arrd.1977.116.4.589 921049

[B12] Bafort J. Timperman G. (1969). Comparative Study of a Generation of Mice Resistant to Plasmodium Berghei. Z. Tropenmed. Parasitol. 20 (1), 74–80. 5380492

[B13] Bakhubaira S. (2013). Hematological Parameters in Severe Complicated Plasmodium Falciparum Malaria Among Adults in Aden. Turk J. Haematol. 30 (4), 394–399. doi: 10.4274/Tjh.2012.0086 24385830 PMC3874965

[B14] Barber B. E. William T. Grigg M. J. Menon J. Auburn S. Marfurt J.. (2013). A Prospective Comparative Study of Knowlesi, Falciparum, and Vivax Malaria in Sabah, Malaysia: High Proportion With Severe Disease From Plasmodium Knowlesi and Plasmodium Vivax But No Mortality With Early Referral and Artesunate Therapy. Clin. Infect. Dis. 56 (3), 383–397. doi: 10.1093/cid/cis902 23087389

[B15] Bartoloni A. Zammarchi L. (2012). Clinical Aspects of Uncomplicated and Severe Malaria. Mediterr. J. Hematol. Infect. Dis. 4 (1), e2012026. doi: 10.4084/MJHID.2012.026 22708041 PMC3375727

[B16] Beckers C. M. van Hinsbergh V. W. van Nieuw Amerongen G. P. (2010). Driving Rho GTPase Activity in Endothelial Cells Regulates Barrier Integrity. Thromb. Haemost. 103 (1), 40–55. doi: 10.1160/TH09-06-0403 20062930

[B17] Belnoue E. Potter S. M. Rosa D. S. Mauduit M. Gruner A. C. Kayibanda M.. (2008). Control of Pathogenic CD8+ T Cell Migration to the Brain by IFN-Gamma During Experimental Cerebral Malaria. Parasite Immunol. 30 (10), 544–553. doi: 10.1111/j.1365-3024.2008.01053.x 18665903

[B18] Bernabeu M. Lopez F. J. Ferrer M. Martin-Jaular L. Razaname A. Corradin G.. (2012). Functional Analysis of Plasmodium Vivax VIR Proteins Reveals Different Subcellular Localizations and Cytoadherence to the ICAM-1 Endothelial Receptor. Cell Microbiol. 14 (3), 386–400. doi: 10.1111/j.1462-5822.2011.01726.x 22103402

[B19] Bosmann M. Ward P. A. (2012). Role of C3, C5 and Anaphylatoxin Receptors in Acute Lung Injury and in Sepsis. Adv. Exp. Med. Biol. 946, 147–159. doi: 10.1007/978-1-4614-0106-3_9 21948367 PMC3372066

[B20] Brasil P. Zalis M. G. de Pina-Costa A. Siqueira A. M. Junior C. B. Silva S.. (2017). Outbreak of Human Malaria Caused by Plasmodium Simium in the Atlantic Forest in Rio De Janeiro: A Molecular Epidemiological Investigation. Lancet Glob. Health 5 (10), e1038–e1046. doi: 10.1016/S2214-109X(17)30333-9 28867401

[B21] Butt Y. Kurdowska A. Allen T. C. (2016). Acute Lung Injury: A Clinical and Molecular Review. Arch. Pathol. Lab. Med. 140 (4), 345–350. doi: 10.5858/arpa.2015-0519-RA 27028393

[B22] Carvalho L. J. Lenzi H. L. Pelajo-Machado M. Oliveira D. N. Daniel-Ribeiro C. T. Ferreira-da-Cruz M. F.. (2000). Plasmodium berghei: Cerebral Malaria in CBA Mice Is Not Clearly Related to Plasma TNF Levels or Intensity of Histopathological Changes. Exp. Parasitol. 95 (1), 1–7. doi: 10.1006/expr.2000.4508 10864512

[B23] Carvalho B. O. Lopes S. C. Nogueira P. A. Orlandi P. P. Bargieri D. Y. Blanco Y. C.. (2010). On the Cytoadhesion of Plasmodium Vivax-Infected Erythrocytes. J. Infect. Dis. 202 (4), 638–647. doi: 10.1086/654815 20617923

[B24] Chang W. L. Jones S. P. Lefer D. J. Welbourne T. Sun G. Yin L.. (2001). CD8(+)-T-Cell Depletion Ameliorates Circulatory Shock in Plasmodium Berghei-Infected Mice. Infect. Immun. 69 (12), 7341–7348. doi: 10.1128/IAI.69.12.7341-7348.2001 11705906 PMC98820

[B25] Chao W. Olson M. S. (1993). Platelet-Activating Factor: Receptors and Signal Transduction. Biochem. J. 292 ( Pt 3), 617–629. doi: 10.1042/bj2920617 8391253 PMC1134157

[B26] Claesson-Welsh L. (2015). Vascular Permeability–the Essentials. Ups J. Med. Sci. 120 (3), 135–143. doi: 10.3109/03009734.2015.1064501 26220421 PMC4526869

[B27] Claser C. Malleret B. Peng K. Bakocevic N. Gun S. Y. Russell B.. (2014). Rodent Plasmodium-Infected Red Blood Cells: Imaging Their Fates and Interactions Within Their Hosts. Parasitol. Int. 63 (1), 187–194. doi: 10.1016/j.parint.2013.07.012 23892178

[B28] Claser C. Nguee S. Y. T. Balachander A. Wu Howland S. Becht E. Gunasegaran B.. (2019). Lung Endothelial Cell Antigen Cross-Presentation to CD8(+)T Cells Drives Malaria-Associated Lung Injury. Nat. Commun. 10 (1), 4241. doi: 10.1038/s41467-019-12017-8 31534124 PMC6751193

[B29] Combes V. Rosenkranz A. R. Redard M. Pizzolato G. Lepidi H. Vestweber D.. (2004). Pathogenic Role of P-Selectin in Experimental Cerebral Malaria: Importance of the Endothelial Compartment. Am. J. Pathol. 164 (3), 781–786. doi: 10.1016/S0002-9440(10)63166-5 14982832 PMC1613268

[B30] Cox-Singh J. Singh B. Daneshvar C. Planche T. Parker-Williams J. Krishna S. (2011). Anti-Inflammatory Cytokines Predominate in Acute Human Plasmodium Knowlesi Infections. PloS One 6 (6), e20541. doi: 10.1371/journal.pone.0020541 21687657 PMC3110641

[B31] Craig A. G. Khairul M. F. Patil P. R. (2012). Cytoadherence and Severe Malaria. Malays J. Med. Sci. 19 (2), 5–18. 22973133 PMC3431742

[B32] Daneshvar C. Davis T. M. Cox-Singh J. Rafa'ee M. Z. Zakaria S. K. Divis P. C.. (2009). Clinical and Laboratory Features of Human Plasmodium Knowlesi Infection. Clin. Infect. Dis. 49 (6), 852–860. doi: 10.1086/605439 19635025 PMC2843824

[B33] Darling T. K. Schenk M. P. Zhou C. C. Maloba F. M. Mimche P. N. Gibbins J. M.. (2020). Platelet Alpha-Granules Contribute to Organ-Specific Pathologies in a Mouse Model of Severe Malaria. Blood Adv. 4 (1), 1–8. doi: 10.1182/bloodadvances.2019000773 31891656 PMC6960474

[B34] David P. H. Hommel M. Miller L. H. Udeinya I. J. Oligino L. D. (1983). Parasite Sequestration in Plasmodium Falciparum Malaria: Spleen and Antibody Modulation of Cytoadherence of Infected Erythrocytes. Proc. Natl. Acad. Sci. U. S. A. 80 (16), 5075–5079. doi: 10.1073/pnas.80.16.5075 6348780 PMC384191

[B35] Day N. P. Hien T. T. Schollaardt T. Loc P. P. Chuong L. V. Chau T. T.. (1999). The Prognostic and Pathophysiologic Role of Pro- and Antiinflammatory Cytokines in Severe Malaria. J. Infect. Dis. 180 (4), 1288–1297. doi: 10.1086/315016 10479160

[B36] de Azevedo-Quintanilha I. G. Medeiros-de-Moraes I. M. Ferreira A. C. Reis P. A. Vieira-de-Abreu A. Campbell R. A.. (2020). Haem Oxygenase Protects Against Thrombocytopaenia and Malaria-Associated Lung Injury. Malar. J. 19 (1), 234. doi: 10.1186/s12936-020-03305-6 32611348 PMC7327213

[B37] de Azevedo-Quintanilha I. G. Vieira-de-Abreu A. Ferreira A. C. Nascimento D. O. Siqueira A. M. Campbell R. A.. (2016). Integrin AlphaDbeta2 (CD11d/CD18) Mediates Experimental Malaria-Associated Acute Respiratory Distress Syndrome (MA-ARDS). Malar. J. 15 (1), 393. doi: 10.1186/s12936-016-1447-7 27473068 PMC4967320

[B38] Dejana E. Bazzoni G. Lampugnani M. G. (1999). Vascular Endothelial (VE)-Cadherin: Only an Intercellular Glue? Exp. Cell Res. 252 (1), 13–19. doi: 10.1006/excr.1999.4601 10502395

[B39] de Jong G. M. Slager J. J. Verbon A. van Hellemond J. J. van Genderen P. J. (2016). Systematic Review of the Role of Angiopoietin-1 and Angiopoietin-2 in Plasmodium Species Infections: Biomarkers or Therapeutic Targets? Malar. J. 15 (1), 581. doi: 10.1186/s12936-016-1624-8 27905921 PMC5134107

[B40] del Portillo H. A. Fernandez-Becerra C. Bowman S. Oliver K. Preuss M. Sanchez C. P.. (2001). A Superfamily of Variant Genes Encoded in the Subtelomeric Region of Plasmodium Vivax. Nature 410 (6830), 839–842. doi: 10.1038/35071118 11298455

[B41] de Mast Q. de Groot P. G. van Heerde W. L. Roestenberg M. van Velzen J. F. Verbruggen B.. (2010). Thrombocytopenia in Early Malaria Is Associated With GP1b Shedding in Absence of Systemic Platelet Activation and Consumptive Coagulopathy. Br. J. Haematol. 151 (5), 495–503. doi: 10.1111/j.1365-2141.2010.08399.x 20955404

[B42] De Niz M. Ullrich A. K. Heiber A. Blancke Soares A. Pick C. Lyck R.. (2016). The Machinery Underlying Malaria Parasite Virulence Is Conserved Between Rodent and Human Malaria Parasites. Nat. Commun. 7, 11659. doi: 10.1038/ncomms11659 27225796 PMC4894950

[B43] Deroost K. Tyberghein A. Lays N. Noppen S. Schwarzer E. Vanstreels E.. (2013). Hemozoin Induces Lung Inflammation and Correlates With Malaria-Associated Acute Respiratory Distress Syndrome. Am. J. Respir. Cell Mol. Biol. 48 (5), 589–600. doi: 10.1165/rcmb.2012-0450OC 23328641

[B44] Desowitz R. S. Miller L. H. Buchanan R. D. Permpanich B. (1969). The Sites of Deep Vascular Schizogony in Plasmodium Coatneyi Malaria. Trans. R. Soc. Trop. Med. Hygiene 63 (2), 198–202. doi: 10.1016/0035-9203(69)90147-3 4978466

[B45] Dole V. S. Bergmeier W. Mitchell H. A. Eichenberger S. C. Wagner D. D. (2005). Activated Platelets Induce Weibel-Palade-Body Secretion and Leukocyte Rolling *In Vivo*: Role of P-Selectin. Blood 106 (7), 2334–2339. doi: 10.1182/blood-2005-04-1530 15956287 PMC1895274

[B46] Dolinay T. Kim Y. S. Howrylak J. Hunninghake G. M. An C. H. Fredenburgh L.. (2012). Inflammasome-Regulated Cytokines Are Critical Mediators of Acute Lung Injury. Am. J. Respir. Crit. Care Med. 185 (11), 1225–1234. doi: 10.1164/rccm.201201-0003OC 22461369 PMC3373064

[B47] Dos Santos Ortolan L. Sercundes M. K. Moura G. C. de Castro Quirino T. Debone D. de Sousa Costa D.. (2019). Endothelial Protein C Receptor Could Contribute to Experimental Malaria-Associated Acute Respiratory Distress Syndrome. J. Immunol. Res. 2019, 3105817. doi: 10.1155/2019/3105817 31871954 PMC6913256

[B48] Duarte M. I. Corbett C. E. Boulos M. Amato Neto V. (1985). Ultrastructure of the Lung in Falciparum Malaria. Am. J. Trop. Med. Hyg. 34 (1), 31–35. doi: 10.4269/ajtmh.1985.34.31 3882010

[B49] Dzeing-Ella A. Nze Obiang P. C. Tchoua R. Planche T. Mboza B. Mbounja M.. (2005). Severe Falciparum Malaria in Gabonese Children: Clinical and Laboratory Features. Malar. J. 4, 1. doi: 10.1186/1475-2875-4-1 15638948 PMC546207

[B50] El-Assaad F. Wheway J. Mitchell A. J. Lou J. Hunt N. H. Combes V.. (2013). Cytoadherence of Plasmodium Berghei-Infected Red Blood Cells to Murine Brain and Lung Microvascular Endothelial Cells *In Vitro* . Infect. Immun. 81 (11), 3984–3991. doi: 10.1128/IAI.00428-13 23940206 PMC3811819

[B51] Elzein F. Mohammed N. Ali N. Bahloul A. Albadani A. Alsherbeeni N. (2017). Pulmonary Manifestation of Plasmodium Falciparum Malaria: Case Reports and Review of the Literature. Respir. Med. Case Rep. 22, 83–86. doi: 10.1016/j.rmcr.2017.06.014 28702342 PMC5496505

[B52] Engwerda C. Belnoue E. Gruner A. C. Renia L. (2005). Experimental Models of Cerebral Malaria. Curr. Top. Microbiol. Immunol. 297, 103–143. 16265904

[B53] Epiphanio S. Campos M. G. Pamplona A. Carapau D. Pena A. C. Ataide R.. (2010). VEGF Promotes Malaria-Associated Acute Lung Injury in Mice. PloS Pathog. 6 (5), e1000916. doi: 10.1371/journal.ppat.1000916 20502682 PMC2873913

[B54] Esamai F. Ernerudh J. Janols H. Welin S. Ekerfelt C. Mining S.. (2003). Cerebral Malaria in Children: Serum and Cerebrospinal Fluid TNF-Alpha and TGF-Beta Levels and Their Relationship to Clinical Outcome. J. Trop. Pediatr. 49 (4), 216–223. doi: 10.1093/tropej/49.4.216 12929882

[B55] Faille D. Combes V. Mitchell A. J. Fontaine A. Juhan-Vague I. Alessi M. C.. (2009). Platelet Microparticles: A New Player in Malaria Parasite Cytoadherence to Human Brain Endothelium. FASEB J. 23 (10), 3449–3458. doi: 10.1096/fj.09-135822 19535685

[B56] Fatih F. A. Siner A. Ahmed A. Woon L. C. Craig A. G. Singh B.. (2012). Cytoadherence and Virulence - The Case of Plasmodium Knowlesi Malaria. Malar. J. 11, 33. doi: 10.1186/1475-2875-11-33 22305466 PMC3330018

[B57] Fauconnier M. Palomo J. Bourigault M. L. Meme S. Szeremeta F. Beloeil J. C.. (2012). IL-12Rbeta2 Is Essential for the Development of Experimental Cerebral Malaria. J. Immunol. 188 (4), 1905–1914. doi: 10.4049/jimmunol.1101978 22238458

[B58] Favre N. Da Laperousaz C. Ryffel B. Weiss N. A. Imhof B. A. Rudin W.. (1999). Role of ICAM-1 (CD54) in the Development of Murine Cerebral Malaria. Microbes Infect. 1 (12), 961–968. doi: 10.1016/s1286-4579(99)80513-9 10617927

[B59] Fernandez-Becerra C. Bernabeu M. Castellanos A. Correa B. R. Obadia T. Ramirez M.. (2020). Plasmodium Vivax Spleen-Dependent Genes Encode Antigens Associated With Cytoadhesion and Clinical Protection. Proc. Natl. Acad. Sci. U. S. A. 117 (23), 13056–13065. doi: 10.1073/pnas.1920596117 32439708 PMC7293605

[B60] Fonager J. Pasini E. M. Braks J. A. Klop O. Ramesar J. Remarque E. J.. (2012). Reduced CD36-Dependent Tissue Sequestration of Plasmodium-Infected Erythrocytes Is Detrimental to Malaria Parasite Growth *In Vivo* . J. Exp. Med. 209 (1), 93–107. doi: 10.1084/jem.20110762 22184632 PMC3260870

[B61] Franke-Fayard B. Fonager J. Braks A. Khan S. M. Janse C. J. (2010). Sequestration and Tissue Accumulation of Human Malaria Parasites: Can We Learn Anything From Rodent Models of Malaria? PLoS Pathog. 6 (9), e1001032. doi: 10.1371/journal.ppat.1001032 20941396 PMC2947991

[B62] Franke-Fayard B. Janse C. J. Cunha-Rodrigues M. Ramesar J. Buscher P. Que I.. (2005). Murine Malaria Parasite Sequestration: CD36 Is the Major Receptor, But Cerebral Pathology Is Unlinked to Sequestration. Proc. Natl. Acad. Sci. U. S. A. 102 (32), 11468–11473. doi: 10.1073/pnas.0503386102 16051702 PMC1183563

[B63] Fazalul Rahiman S. S. Basir R. Talib H. Tie T. H. Chuah Y. K. Jabbarzare M.. (2013). Interleukin-27 Exhibited Anti-Inflammatory Activity During Plasmodium berghei Infection in Mice. Trop. Biomed. 30 (4), 663–680 24522137

[B64] Fu Y. Ding Y. Zhou T. L. Ou Q. Y. Xu W. Y. (2012). Comparative Histopathology of Mice Infected With the 17XL and 17XNL Strains of Plasmodium yoelii. J. Parasitol. 98 (2), 310–315. doi: 10.1645/GE-2825.1 22017443

[B65] Fujita M. Kuwano K. Kunitake R. Hagimoto N. Miyazaki H. Kaneko Y.. (1998). Endothelial Cell Apoptosis in Lipopolysaccharide-Induced Lung Injury in Mice. Int. Arch. Allergy Immunol. 117 (3), 202–208. doi: 10.1159/000024011 9831808

[B66] Gallego-Delgado J. Basu-Roy U. Ty M. Alique M. Fernandez-Arias C. Movila A.. (2016). Angiotensin Receptors and Beta-Catenin Regulate Brain Endothelial Integrity in Malaria. J. Clin. Invest. 126 (10), 4016–4029. doi: 10.1172/JCI87306 27643439 PMC5096829

[B67] Galvao-Filho B. de Castro J. T. Figueiredo M. M. Rosmaninho C. G. Antonelli L. Gazzinelli R. T. (2019). The Emergence of Pathogenic TNF/iNOS Producing Dendritic Cells (Tip-DCs) in a Malaria Model of Acute Respiratory Distress Syndrome (ARDS) Is Dependent on CCR4. Mucosal Immunol. 12 (2), 312–322. doi: 10.1038/s41385-018-0093-5 30337650 PMC6375779

[B68] Gerardin P. Rogier C. Ka A. S. Jouvencel P. Brousse V. Imbert P. (2002). Prognostic Value of Thrombocytopenia in African Children With Falciparum Malaria. Am. J. Trop. Med. Hyg. 66 (6), 686–691. doi: 10.4269/ajtmh.2002.66.686 12224575

[B69] Gillrie M. R. Krishnegowda G. Lee K. Buret A. G. Robbins S. M. Looareesuwan S.. (2007). Src-Family Kinase Dependent Disruption of Endothelial Barrier Function by Plasmodium Falciparum Merozoite Proteins. Blood 110 (9), 3426–3435. doi: 10.1182/blood-2007-04-084582 17693580 PMC2200906

[B70] Goggi J. L. Claser C. Hartimath S. V. Hor P. X. Tan P. W. Ramasamy B.. (2021). PET Imaging of Translocator Protein as a Marker of Malaria-Associated Lung Inflammation. Infect. Immun. 89 (10), e0002421. doi: 10.1128/IAI.00024-21 34251290 PMC8445173

[B71] Graham S. M. Chen J. Chung D. W. Barker K. R. Conroy A. L. Hawkes M. T.. (2016). Endothelial Activation, Haemostasis and Thrombosis Biomarkers in Ugandan Children With Severe Malaria Participating in a Clinical Trial. Malar. J. 15, 56. doi: 10.1186/s12936-016-1106-z 26830467 PMC4736470

[B72] Grau G. E. Fajardo L. F. Piguet P. F. Allet B. Lambert P. H. Vassalli P. (1987). Tumor Necrosis Factor (Cachectin) as an Essential Mediator in Murine Cerebral Malaria. Science 237 (4819), 1210–1212. doi: 10.1126/science.3306918 3306918

[B73] Groger M. Fischer H. S. Veletzky L. Lalremruata A. Ramharter M. (2017). A Systematic Review of the Clinical Presentation, Treatment and Relapse Characteristics of Human Plasmodium Ovale Malaria. Malar. J. 16 (1), 112. doi: 10.1186/s12936-017-1759-2 28284211 PMC5346189

[B74] Guido B. C. Zanatelli M. Tavares-de-Lima W. Oliani S. M. Damazo A. S. (2013). Annexin-A1 Peptide Down-Regulates the Leukocyte Recruitment and Up-Regulates Interleukin-10 Release Into Lung After Intestinal Ischemia-Reperfusion in Mice. J. Inflamm. (Lond) 10 (1), 10. doi: 10.1186/1476-9255-10-10 23497133 PMC3608250

[B75] Guillon A. Arafa E. I. Barker K. A. Belkina A. C. Martin I. Shenoy A. T.. (2020). Pneumonia Recovery Reprograms the Alveolar Macrophage Pool. JCI Insight 5 (4), 1–20. doi: 10.1172/jci.insight.133042 PMC7101156 31990682

[B76] Gun S. Y. Claser C. Tan K. S. Renia L. (2014). Interferons and Interferon Regulatory Factors in Malaria. Mediators Inflamm. 2014, 243713. doi: 10.1155/2014/243713 25157202 PMC4124246

[B77] Hansen D. S. Evans K. J. D'Ombrain M. C. Bernard N. J. Sexton A. C. Buckingham L.. (2005). The Natural Killer Complex Regulates Severe Malarial Pathogenesis and Influences Acquired Immune Responses to Plasmodium Berghei ANKA. Infect. Immun. 73 (4), 2288–2297. doi: 10.1128/IAI.73.4.2288-2297.2005 15784573 PMC1087422

[B78] Hartl D. Griese M. Nicolai T. Zissel G. Prell C. Reinhardt D.. (2005). A Role for MCP-1/CCR2 in Interstitial Lung Disease in Children. Respir. Res. 6, 93. doi: 10.1186/1465-9921-6-93 16095529 PMC1199626

[B79] Hartmann S. Ridley A. J. Lutz S. (2015). The Function of Rho-Associated Kinases ROCK1 and ROCK2 in the Pathogenesis of Cardiovascular Disease. Front. Pharmacol. 6, 276. doi: 10.3389/fphar.2015.00276 26635606 PMC4653301

[B80] Haydoura S. Mazboudi O. Charafeddine K. Bouakl I. Baban T. A. Taher A. T.. (2011). Transfusion-Related Plasmodium Ovale Malaria Complicated by Acute Respiratory Distress Syndrome (ARDS) in a Non-Endemic Country. Parasitol. Int. 60 (1), 114–116. doi: 10.1016/j.parint.2010.10.005 20971212

[B81] Hee L. Dinudom A. Mitchell A. J. Grau G. E. Cook D. I. Hunt N. H.. (2011). Reduced Activity of the Epithelial Sodium Channel in Malaria-Induced Pulmonary Oedema in Mice. Int. J. Parasitol. 41 (1), 81–88. doi: 10.1016/j.ijpara.2010.07.013 20816846 PMC7125784

[B82] Heil M. Clauss M. Suzuki K. Buschmann I. R. Willuweit A. Fischer S.. (2000). Vascular Endothelial Growth Factor (VEGF) Stimulates Monocyte Migration Through Endothelial Monolayers *via* Increased Integrin Expression. Eur. J. Cell Biol. 79 (11), 850–857. doi: 10.1078/0171-9335-00113 11139149

[B83] Henry B. Roussel C. Carucci M. Brousse V. Ndour P. A. Buffet P. (2020). The Human Spleen in Malaria: Filter or Shelter? Trends Parasitol. 36 (5), 435–446. doi: 10.1016/j.pt.2020.03.001 32298631

[B84] He Y. Zhang Y. Wu H. Luo J. Cheng C. Zhang H. (2021). The Role of Annexin A1 Peptide in Regulating PI3K/Akt Signaling Pathway to Reduce Lung Injury After Cardiopulmonary Bypass in Rats. Perfusion 2676591211052162. doi: 10.1177/02676591211052162 34951334

[B85] Hu G. Ye R. D. Dinauer M. C. Malik A. B. Minshall R. D. (2008). Neutrophil Caveolin-1 Expression Contributes to Mechanism of Lung Inflammation and Injury. Am. J. Physiol. Lung Cell Mol. Physiol. 294 (2), L178–L186. doi: 10.1152/ajplung.00263.2007 17993589

[B86] Jambou R. Combes V. Jambou M. J. Weksler B. B. Couraud P. O. Grau G. E. (2010). Plasmodium Falciparum Adhesion on Human Brain Microvascular Endothelial Cells Involves Transmigration-Like Cup Formation and Induces Opening of Intercellular Junctions. PloS Pathog. 6 (7), e1001021. doi: 10.1371/journal.ppat.1001021 20686652 PMC2912387

[B87] Janssen C. S. Phillips R. S. Turner C. M. Barrett M. P. (2004). Plasmodium Interspersed Repeats: The Major Multigene Superfamily of Malaria Parasites. Nucleic Acids Res. 32 (19), 5712–5720. doi: 10.1093/nar/gkh907 15507685 PMC528792

[B88] Ji W. Hu Q. Zhang M. Zhang C. Chen C. Yan Y.. (2021). The Disruption of the Endothelial Barrier Contributes to Acute Lung Injury Induced by Coxsackievirus A2 Infection in Mice. Int. J. Mol. Sci. 22 (18), 1–16. doi: 10.3390/ijms22189895 PMC8467819 34576058

[B89] Joyner J. C. Consortium T.M Wood J. S. Moreno A. Garcia A. Galinski M. R. (2017). Case Report: Severe and Complicated Cynomolgi Malaria in a Rhesus Macaque Resulted in Similar Histopathological Changes as Those Seen in Human Malaria. Am. J. Trop. Med. Hyg. 97 (2), 548–555. doi: 10.4269/ajtmh.16-0742 28829738 PMC5544077

[B90] Ju Y. Qiu L. Sun X. Liu H. Gao W. (2021). Ac2-26 Mitigated Acute Respiratory Distress Syndrome Rats *via* Formyl Peptide Receptor Pathway. Ann. Med. 53 (1), 653–661. doi: 10.1080/07853890.2021.1925149 34008449 PMC8143635

[B91] Karunaweera N. D. Grau G. E. Gamage P. Carter R. Mendis K. N. (1992). Dynamics of Fever and Serum Levels of Tumor Necrosis Factor Are Closely Associated During Clinical Paroxysms in Plasmodium Vivax Malaria. Proc. Natl. Acad. Sci. U. S. A. 89 (8), 3200–3203. doi: 10.1073/pnas.89.8.3200 1565611 PMC48833

[B92] Karunaweera N. D. Wijesekera S. K. Wanasekera D. Mendis K. N. Carter R. (2003). The Paroxysm of Plasmodium Vivax Malaria. Trends Parasitol. 19 (4), 188–193. doi: 10.1016/s1471-4922(03)00036-9 12689650

[B93] Kim H. Higgins S. Liles W. C. Kain K. C. (2011). Endothelial Activation and Dysregulation in Malaria: A Potential Target for Novel Therapeutics. Curr. Opin. Hematol. 18 (3), 177–185. doi: 10.1097/MOH.0b013e328345a4cf 21423010

[B94] King T. Lamb T. (2015). Interferon-Gamma: The Jekyll and Hyde of Malaria. PloS Pathog. 11 (10), e1005118. doi: 10.1371/journal.ppat.1005118 26426121 PMC4591348

[B95] Kingston H. W. F. Ghose A. Rungpradubvong V. Satitthummanid S. Herdman M. T. Plewes K.. (2020). Cell-Free Hemoglobin Is Associated With Increased Vascular Resistance and Reduced Peripheral Perfusion in Severe Malaria. J. Infect. Dis. 221 (1), 127–137. doi: 10.1093/infdis/jiz359 31693729

[B96] Kinra P. Dutta V. (2013). Serum TNF Alpha Levels: A Prognostic Marker for Assessment of Severity of Malaria. Trop. BioMed. 30 (4), 645–653. 24522135

[B97] Knackstedt S. L. Georgiadou A. Apel F. Abu-Abed U. Moxon C. A. Cunnington A. J.. (2019). Neutrophil Extracellular Traps Drive Inflammatory Pathogenesis in Malaria. Sci. Immunol. 4 (40), 1–17. doi: 10.1126/sciimmunol.aaw0336 PMC6892640 31628160

[B98] Kobayashi T. Tanaka K. Fujita T. Umezawa H. Amano H. Yoshioka K.. (2015). Bidirectional Role of IL-6 Signal in Pathogenesis of Lung Fibrosis. Respir. Res. 16, 99. doi: 10.1186/s12931-015-0261-z 26289430 PMC4546032

[B99] Korir C. C. Galinski M. R. (2006). Proteomic Studies of Plasmodium Knowlesi SICA Variant Antigens Demonstrate Their Relationship With P. Falciparum EMP1. Infect. Genet. Evol. 6 (1), 75–79. doi: 10.1016/j.meegid.2005.01.003 16376842

[B100] Kraisin S. Verhenne S. Pham T. T. Martinod K. Tersteeg C. Vandeputte N.. (2019). Von Willebrand Factor in Experimental Malaria-Associated Acute Respiratory Distress Syndrome. J. Thromb. Haemost. 17 (8), 1372–1383. doi: 10.1111/jth.14485 31099973 PMC9906160

[B101] Kremsner P. G. Neifer S. Chaves M. F. Rudolph R. Bienzle U. (1992). Interferon-Gamma Induced Lethality in the Late Phase of Plasmodium vinckei Malaria Despite Effective Parasite Clearance by Chloroquine. Eur. J. Immunol. 22 (11), 2873–2878. doi: 10.1002/eji.1830221118 1425913

[B102] Krishnamurthy V. R. Sardar M. Y. Ying Y. Song X. Haller C. Dai E.. (2015). Glycopeptide Analogues of PSGL-1 Inhibit P-Selectin *In Vitro* and *In Vivo* . Nat. Commun. 6, 6387. doi: 10.1038/ncomms7387 25824568 PMC4423566

[B103] Krishna S. Waller D. W. ter Kuile F. Kwiatkowski D. Crawley J. Craddock C. F.. (1994). Lactic Acidosis and Hypoglycaemia in Children With Severe Malaria: Pathophysiological and Prognostic Significance. Trans. R. Soc. Trop. Med. Hyg. 88 (1), 67–73. doi: 10.1016/0035-9203(94)90504-5 8154008

[B104] Lacerda M. V. Fragoso S. C. Alecrim M. G. Alexandre M. A. Magalhaes B. M. Siqueira A. M.. (2012). Postmortem Characterization of Patients With Clinical Diagnosis of Plasmodium Vivax Malaria: To What Extent Does This Parasite Kill? Clin. Infect. Dis. 55 (8), e67–e74. doi: 10.1093/cid/cis615 22772803

[B105] Lacerda M. V. Mourao M. P. Coelho H. C. Santos J. B. (2011). Thrombocytopenia in Malaria: Who Cares? Mem. Inst. Oswaldo Cruz 106 Suppl 1, 52–63. doi: 10.1590/s0074-02762011000900007 21881757

[B106] Lacerda-Queiroz N. Rachid M. A. Teixeira M. M. Teixeira A. L. (2013). The Role of Platelet-Activating Factor Receptor (PAFR) in Lung Pathology During Experimental Malaria. Int. J. Parasitol. 43 (1), 11–15. doi: 10.1016/j.ijpara.2012.11.008 23260771

[B107] Ladhani S. Lowe B. Cole A. O. Kowuondo K. Newton C. R. (2002). Changes in White Blood Cells and Platelets in Children With Falciparum Malaria: Relationship to Disease Outcome. Br. J. Haematol. 119 (3), 839–847. doi: 10.1046/j.1365-2141.2002.03904.x 12437669

[B108] Lagasse H. A. Anidi I. U. Craig J. M. Limjunyawong N. Poupore A. K. Mitzner W.. (2016). Recruited Monocytes Modulate Malaria-Induced Lung Injury Through CD36-Mediated Clearance of Sequestered Infected Erythrocytes. J. Leukoc. Biol. 99 (5), 659–671. doi: 10.1189/jlb.4HI0315-130RRR 26516185 PMC6608510

[B109] Lalremruata A. Magris M. Vivas-Martinez S. Koehler M. Esen M. Kempaiah P.. (2015). Natural Infection of Plasmodium Brasilianum in Humans: Man and Monkey Share Quartan Malaria Parasites in the Venezuelan Amazon. EBioMedicine 2 (9), 1186–1192. doi: 10.1016/j.ebiom.2015.07.033 26501116 PMC4588399

[B110] Lawand M. Dechanet-Merville J. Dieu-Nosjean M. C. (2017). Key Features of Gamma-Delta T-Cell Subsets in Human Diseases and Their Immunotherapeutic Implications. Front. Immunol. 8, 761. doi: 10.3389/fimmu.2017.00761 28713381 PMC5491929

[B111] Lee W. C. Russell B. Renia L. (2019). Sticking for a Cause: The Falciparum Malaria Parasites Cytoadherence Paradigm. Front. Immunol. 10, 1444. doi: 10.3389/fimmu.2019.01444 31316507 PMC6610498

[B112] Lee W. C. Shahari S. Nguee S. Y. T. Lau Y. L. Renia L. (2021). Cytoadherence Properties of Plasmodium Knowlesi-Infected Erythrocytes. Front. Microbiol. 12, 4417. doi: 10.3389/fmicb.2021.804417 PMC8767020 35069511

[B113] Le T. T. Karmouty-Quintana H. Melicoff E. Le T. T. Weng T. Chen N. Y.. (2014). Blockade of IL-6 Trans Signaling Attenuates Pulmonary Fibrosis. J. Immunol. 193 (7), 3755–3768. doi: 10.4049/jimmunol.1302470 25172494 PMC4169999

[B114] Li J. Chang W. L. Sun G. Chen H. L. Specian R. D. Berney S. M.. (2003). Intercellular Adhesion Molecule 1 Is Important for the Development of Severe Experimental Malaria But Is Not Required for Leukocyte Adhesion in the Brain. J. Investig. Med. 51 (3), 128–140. doi: 10.1136/jim-51-03-15 12769195

[B115] Liew J. W. K. Bukhari F. D. M. Jeyaprakasam N. K. Phang W. K. Vythilingam I. Lau Y. L. (2021). Natural Plasmodium Inui Infections in Humans and Anopheles Cracens Mosquito, Malaysia. Emerg. Infect. Dis. 27 (10), 2700–2703. doi: 10.3201/eid2710.210412 34545786 PMC8462313

[B116] Lin J. W. Sodenkamp J. Cunningham D. Deroost K. Tshitenge T. C. McLaughlin S.. (2017). Signatures of Malaria-Associated Pathology Revealed by High-Resolution Whole-Blood Transcriptomics in a Rodent Model of Malaria. Sci. Rep. 7, 41722. doi: 10.1038/srep41722 28155887 PMC5290525

[B117] Liu M. Amodu A. S. Pitts S. Patrickson J. Hibbert J. M. Battle M.. (2012). Heme Mediated STAT3 Activation in Severe Malaria. PloS One 7 (3), e34280. doi: 10.1371/journal.pone.0034280 22479586 PMC3316628

[B118] Liu M. Gu C. Wang Y. (2014). Upregulation of the Tight Junction Protein Occludin: Effects on Ventilation-Induced Lung Injury and Mechanisms of Action. BMC Pulm. Med. 14, 94. doi: 10.1186/1471-2466-14-94 24884662 PMC4046497

[B119] Lopes S. C. Albrecht L. Carvalho B. O. Siqueira A. M. Thomson-Luque R. Nogueira P. A.. (2014). Paucity of Plasmodium Vivax Mature Schizonts in Peripheral Blood Is Associated With Their Increased Cytoadhesive Potential. J. Infect. Dis. 209 (9), 1403–1407. doi: 10.1093/infdis/jiu018 24415786

[B120] Lovegrove F. E. Gharib S. A. Pena-Castillo L. Patel S. N. Ruzinski J. T. Hughes T. R.. (2008). Parasite Burden and CD36-Mediated Sequestration Are Determinants of Acute Lung Injury in an Experimental Malaria Model. PloS Pathog. 4 (5), e1000068. doi: 10.1371/journal.ppat.1000068 18483551 PMC2364663

[B121] Lozano F. Leal M. Lissen E. Munoz J. Bautista A. Regordan C. (1983). [P. Falciparum and P. Malariae Malaria Complicated by Pulmonary Edema With Disseminated Intravascular Coagulation]. Presse Med. 12 (47), 3004–3005. 6228898

[B122] MacCallum D. K. (1968). Pulmonary Changes Resulting From Experimental Malaria Infection in Hamsters. Arch. Pathol. 86 (6), 681–688. 5701643

[B123] MacCallum D. K. (1969). A Study of Macrophage--Pulmonary Vascular Bed Interactions in Malaria-Infected Hamsters. J. Reticuloendothel. Soc. 6 (3), 253–270. 5790418

[B124] MacPherson G. G. Warrell M. J. White N. J. Looareesuwan S. Warrell D. A. (1985). Human Cerebral Malaria. A Quantitative Ultrastructural Analysis of Parasitized Erythrocyte Sequestration. Am. J. Pathol. 119 (3), 385–401. 3893148 PMC1888001

[B125] Maguire G. P. Handojo T. Pain M. C. Kenangalem E. Price R. N. Tjitra E.. (2005). Lung Injury in Uncomplicated and Severe Falciparum Malaria: A Longitudinal Study in Papua, Indonesia. J. Infect. Dis. 192 (11), 1966–1974. doi: 10.1086/497697 16267769 PMC2566801

[B126] Maknitikul S. Luplertlop N. Grau G. E. R. Ampawong S. (2017). Dysregulation of Pulmonary Endothelial Protein C Receptor and Thrombomodulin in Severe Falciparum Malaria-Associated ARDS Relevant to Hemozoin. PloS One 12 (7), e0181674. doi: 10.1371/journal.pone.0181674 28732053 PMC5521846

[B127] Mandala W. L. Msefula C. L. Gondwe E. N. Drayson M. T. Molyneux M. E. MacLennan C. A. (2017). Cytokine Profiles in Malawian Children Presenting With Uncomplicated Malaria, Severe Malarial Anemia, and Cerebral Malaria. Clin. Vaccine Immunol. 24 (4), 1–11. doi: 10.1128/CVI.00533-16 PMC5382826 28122790

[B128] Marcos-Ramiro B. Garcia-Weber D. Millan J. (2014). TNF-Induced Endothelial Barrier Disruption: Beyond Actin and Rho. Thromb. Haemost. 112 (6), 1088–1102. doi: 10.1160/TH14-04-0299 25078148

[B129] Ma Y. Yang X. Chatterjee V. Meegan J. E. Beard R. S. Jr. Yuan S. Y. (2019). Role of Neutrophil Extracellular Traps and Vesicles in Regulating Vascular Endothelial Permeability. Front. Immunol. 10, 1037. doi: 10.3389/fimmu.2019.01037 31143182 PMC6520655

[B130] McKenzie J. A. Ridley A. J. (2007). Roles of Rho/ROCK and MLCK in TNF-Alpha-Induced Changes in Endothelial Morphology and Permeability. J. Cell Physiol. 213 (1), 221–228. doi: 10.1002/jcp.21114 17476691

[B131] McMorran B. J. Marshall V. M. de Graaf C. Drysdale K. E. Shabbar M. Smyth G. K.. (2009). Platelets Kill Intraerythrocytic Malarial Parasites and Mediate Survival to Infection. Science 323 (5915), 797–800. doi: 10.1126/science.1166296 19197068

[B132] McMorran B. J. Wieczorski L. Drysdale K. E. Chan J. A. Huang H. M. Smith C.. (2012). Platelet Factor 4 and Duffy Antigen Required for Platelet Killing of Plasmodium Falciparum. Science 338 (6112), 1348–1351. doi: 10.1126/science.1228892 23224555

[B133] Meegan J. E. Shaver C. M. Putz N. D. Jesse J. J. Landstreet S. R. Lee H. N. R.. (2020). Cell-Free Hemoglobin Increases Inflammation, Lung Apoptosis, and Microvascular Permeability in Murine Polymicrobial Sepsis. PloS One 15 (2), e0228727. doi: 10.1371/journal.pone.0228727 32012200 PMC6996826

[B134] Mehta D. Malik A. B. (2006). Signaling Mechanisms Regulating Endothelial Permeability. Physiol. Rev. 86 (1), 279–367. doi: 10.1152/physrev.00012.2005 16371600

[B135] Millar F. R. Summers C. Griffiths M. J. Toshner M. R. Proudfoot A. G. (2016). The Pulmonary Endothelium in Acute Respiratory Distress Syndrome: Insights and Therapeutic Opportunities. Thorax 71 (5), 462–473. doi: 10.1136/thoraxjnl-2015-207461 26968969

[B136] Miller L. H. Fremount H. N. Luse S. A. (1971). Deep Vascular Schizogony of Plasmodium Knowlesi in Macaca Mulatta. Distribution in Organs and Ultrastructure of Parasitized Red Cells. Am. J. Trop. Med. Hyg. 20 (6), 816–824. doi: 10.4269/ajtmh.1971.20.816 5002246

[B137] Mills C. D. Kincaid K. Alt J. M. Heilman M. J. Hill A. M. (2000). M-1/M-2 Macrophages and the Th1/Th2 Paradigm. J. Immunol. 164 (12), 6166–6173. doi: 10.4049/jimmunol.164.12.6166 10843666

[B138] Milner D. Jr. Factor R. Whitten R. Carr R. A. Kamiza S. Pinkus G.. (2013). Pulmonary Pathology in Pediatric Cerebral Malaria. Hum. Pathol. 44 (12), 2719–2726. doi: 10.1016/j.humpath.2013.07.018 24074535 PMC3838443

[B139] Mohan A. Sharma S. K. Bollineni S. (2008). Acute Lung Injury and Acute Respiratory Distress Syndrome in Malaria. J. Vector Borne Dis. 45 (3), 179–193. 18807374

[B140] Moore B. R. Jago J. D. Batty K. T. (2008). Plasmodium berghei: Parasite Clearance After Treatment With Dihydroartemisinin in an asplenic Murine Malaria Model. Exp. Parasitol. 118 (4), 458–467. doi: 10.1016/j.exppara.2007.10.011 18023429

[B141] Moxon C. A. Wassmer S. C. Milner D. A. Jr. Chisala N. V. Taylor T. E. Seydel K. B.. (2013). Loss of Endothelial Protein C Receptors Links Coagulation and Inflammation to Parasite Sequestration in Cerebral Malaria in African Children. Blood 122 (5), 842–851. doi: 10.1182/blood-2013-03-490219 23741007 PMC3731936

[B142] Netea M. G. Kullberg B. J. van der Meer J. W. (2000). Circulating Cytokines as Mediators of Fever. Clin. Infect. Dis. 31 Suppl 5, S178–S184. doi: 10.1086/317513 11113021

[B143] Norbert Voelkel S. R. (2009). The Pulmonary Endothelium: Function in Health and Disease (Wiley-Blackwell).

[B144] Ockenhouse C. F. Tegoshi T. Maeno Y. Benjamin C. Ho M. Kan K. E.. (1992). Human Vascular Endothelial Cell Adhesion Receptors for Plasmodium Falciparum-Infected Erythrocytes: Roles for Endothelial Leukocyte Adhesion Molecule 1 and Vascular Cell Adhesion Molecule 1. J. Exp. Med. 176 (4), 1183–1189. doi: 10.1084/jem.176.4.1183 1383378 PMC2119387

[B145] Olliaro P. (2008). Editorial Commentary: Mortality Associated With Severe Plasmodium Falciparum Malaria Increases With Age. Clin. Infect. Dis. 47 (2), 158–160. doi: 10.1086/589288 18564928

[B146] Ortolan L. S. Sercundes M. K. Barboza R. Debone D. Murillo O. Hagen S. C.. (2014). Predictive Criteria to Study the Pathogenesis of Malaria-Associated ALI/ARDS in Mice. Mediators Inflamm. 2014, 872464. doi: 10.1155/2014/872464 25276057 PMC4167651

[B147] Ozwara H. Langermans J. A. Maamun J. Farah I. O. Yole D. S. Mwenda J. M.. (2003). Experimental Infection of the Olive Baboon (Paplio anubis) With Plasmodium knowlesi: Severe Disease Accompanied by Cerebral Involvement. Am. J. Trop. Med. Hyg. 69 (2), 188–194. 13677374

[B148] Pain A. Ferguson D. J. Kai O. Urban B. C. Lowe B. Marsh K.. (2001). Platelet-Mediated Clumping of Plasmodium Falciparum-Infected Erythrocytes Is a Common Adhesive Phenotype and Is Associated With Severe Malaria. Proc. Natl. Acad. Sci. U. S. A. 98 (4), 1805–1810. doi: 10.1073/pnas.98.4.1805 11172032 PMC29338

[B149] Park G. S. Ireland K. F. Opoka R. O. John C. C. (2012). Evidence of Endothelial Activation in Asymptomatic Plasmodium Falciparum Parasitemia and Effect of Blood Group on Levels of Von Willebrand Factor in Malaria. J. Pediatr. Infect. Dis. Soc. 1 (1), 16–25. doi: 10.1093/jpids/pis010 PMC3656549 23687570

[B150] Pedersen S. F. Ho Y. C. (2020). SARS-CoV-2: A Storm Is Raging. J. Clin. Invest. 130 (5), 2202–2205. doi: 10.1172/JCI137647 32217834 PMC7190904

[B151] Peerschke E. I. Yin W. Ghebrehiwet B. (2010). Complement Activation on Platelets: Implications for Vascular Inflammation and Thrombosis. Mol. Immunol. 47 (13), 2170–2175. doi: 10.1016/j.molimm.2010.05.009 20621693 PMC2904326

[B152] Pereira M. L. Ortolan L. S. Sercundes M. K. Debone D. Murillo O. Lima F. A.. (2016). Association of Heme Oxygenase 1 With Lung Protection in Malaria-Associated ALI/ARDS. Mediators Inflamm. 2016, 4158698. doi: 10.1155/2016/4158698 27974865 PMC5126464

[B153] Peterson M. S. Joyner C. J. Brady J. A. Wood J. S. Cabrera-Mora M. Saney C. L.. (2021). Clinical Recovery of Macaca Fascicularis Infected With Plasmodium Knowlesi. Malar. J. 20 (1), 486. doi: 10.1186/s12936-021-03925-6 34969401 PMC8719393

[B154] Peterson M. S. Joyner C. J. Cordy R. J. Salinas J. L. Machiah D. Lapp S. A.. (2019). Plasmodium Vivax Parasite Load Is Associated With Histopathology in Saimiri Boliviensis With Findings Comparable to P Vivax Pathogenesis in Humans. Open Forum Infect. Dis. 6 (3), ofz021. doi: 10.1093/ofid/ofz021 30937329 PMC6436601

[B155] Pham T. T. Punsawad C. Glaharn S. De Meyer S. F. Viriyavejakul P. Van den Steen P. E. (2019). Release of Endothelial Activation Markers in Lungs of Patients With Malaria-Associated Acute Respiratory Distress Syndrome. Malar. J. 18 (1), 395. doi: 10.1186/s12936-019-3040-3 31796023 PMC6891978

[B156] Pham T. T. Verheijen M. Vandermosten L. Deroost K. Knoops S. Van den Eynde K.. (2017). Pathogenic CD8(+) T Cells Cause Increased Levels of VEGF-A in Experimental Malaria-Associated Acute Respiratory Distress Syndrome, But Therapeutic VEGFR Inhibition Is Not Effective. Front. Cell Infect. Microbiol. 7, 416. doi: 10.3389/fcimb.2017.00416 29034214 PMC5627041

[B157] Piguet P. F. Da Laperrousaz C. Vesin C. Tacchini-Cottier F. Senaldi G. Grau G. E. (2000). Delayed Mortality and Attenuated Thrombocytopenia Associated With Severe Malaria in Urokinase- and Urokinase Receptor-Deficient Mice. Infect. Immun. 68 (7), 3822–3829. doi: 10.1128/IAI.68.7.3822-3829.2000 10858190 PMC101654

[B158] Piguet P. F. Kan C. D. Vesin C. (2002). Role of the Tumor Necrosis Factor Receptor 2 (TNFR2) in Cerebral Malaria in Mice. Lab. Invest. 82 (9), 1155–1166. doi: 10.1097/01.lab.0000028822.94883.8a 12218076

[B159] Pollenus E. Pham T. T. Vandermosten L. Possemiers H. Knoops S. Opdenakker G.. (2020). CCR2 Is Dispensable for Disease Resolution But Required for the Restoration of Leukocyte Homeostasis Upon Experimental Malaria-Associated Acute Respiratory Distress Syndrome. Front. Immunol. 11, 628643. doi: 10.3389/fimmu.2020.628643 33664739 PMC7921736

[B160] Possemiers H. Pham T. T. Coens M. Pollenus E. Knoops S. Noppen S.. (2021). Skeleton Binding Protein-1-Mediated Parasite Sequestration Inhibits Spontaneous Resolution of Malaria-Associated Acute Respiratory Distress Syndrome. PloS Pathog. 17 (11), e1010114. doi: 10.1371/journal.ppat.1010114 34843584 PMC8659713

[B161] Price R. N. Tjitra E. Guerra C. A. Yeung S. White N. J. Anstey N. M. (2007). Vivax Malaria: Neglected and Not Benign. Am. J. Trop. Med. Hyg. 77 (6 Suppl), 79–87. doi: 10.4269/ajtmh.2007.77.79 18165478 PMC2653940

[B162] Punsawad C. Viriyavejakul P. Setthapramote C. Palipoch S. (2015). Enhanced Expression of Fas and FasL Modulates Apoptosis in the Lungs of Severe P. Falciparum Malaria Patients With Pulmonary Edema. Int. J. Clin. Exp. Pathol. 8 (9), 10002–10013. 26617708 PMC4637523

[B163] Quirino T. C. Ortolan L. D. S. Sercundes M. K. Marinho C. R. F. Turato W. M. Epiphanio S. (2020). Lung Aeration in Experimental Malaria-Associated Acute Respiratory Distress Syndrome by SPECT/CT Analysis. PloS One 15 (5), e0233864. doi: 10.1371/journal.pone.0233864 32470082 PMC7259762

[B164] Ragab D. Salah Eldin H. Taeimah M. Khattab R. Salem R. (2020). The COVID-19 Cytokine Storm; What We Know So Far. Front. Immunol. 11, 1446. doi: 10.3389/fimmu.2020.01446 32612617 PMC7308649

[B165] Reid P. T. Donnelly S. C. (1996). Predicting Acute Respiratory Distress Syndrome and Intrapulmonary Inflammation. Br. J. Hosp. Med. 55 (8), 499–502. 8732223

[B166] Reinders M. E. Sho M. Izawa A. Wang P. Mukhopadhyay D. Koss K. E.. (2003). Proinflammatory Functions of Vascular Endothelial Growth Factor in Alloimmunity. J. Clin. Invest. 112 (11), 1655–1665. doi: 10.1172/JCI17712 14660742 PMC281640

[B167] Schappo A. P. Bittencourt N. C. Bertolla L. P. Forcellini S. da Silva A. Dos Santos H. G.. (2022). Antigenicity and Adhesiveness of a Plasmodium Vivax VIR-E Protein From Brazilian Isolates. Mem. Inst. Oswaldo Cruz 116, e210227. doi: 10.1590/0074-02760210227 35137905 PMC8824159

[B168] Schneberger D. Aharonson-Raz K. Singh B. (2011). Monocyte and Macrophage Heterogeneity and Toll-Like Receptors in the Lung. Cell Tissue Res. 343 (1), 97–106. doi: 10.1007/s00441-010-1032-2 20824285

[B169] Sercundes M. K. Ortolan L. S. da Silva Julio V. Bella L. M. de Castro Quirino T. Debone D.. (2022). Blockade of Caspase Cascade Overcomes Malaria-Associated Acute Respiratory Distress Syndrome in Mice. Cell Death Dis. 13 (2), 144. doi: 10.1038/s41419-022-04582-6 35145061 PMC8831525

[B170] Sercundes M. K. Ortolan L. S. Debone D. Soeiro-Pereira P. V. Gomes E. Aitken E. H.. (2016). Targeting Neutrophils to Prevent Malaria-Associated Acute Lung Injury/Acute Respiratory Distress Syndrome in Mice. PloS Pathog. 12 (12), e1006054. doi: 10.1371/journal.ppat.1006054 27926944 PMC5142790

[B171] Sharron M. Hoptay C. E. Wiles A. A. Garvin L. M. Geha M. Benton A. S.. (2012). Platelets Induce Apoptosis During Sepsis in a Contact-Dependent Manner That Is Inhibited by GPIIb/IIIa Blockade. PloS One 7 (7), e41549. doi: 10.1371/journal.pone.0041549 22844498 PMC3406039

[B172] Singh B. Kim Sung L. Matusop A. Radhakrishnan A. Shamsul S. S. Cox-Singh J.. (2004). A Large Focus of Naturally Acquired Plasmodium Knowlesi Infections in Human Beings. Lancet 363 (9414), 1017–1024. doi: 10.1016/S0140-6736(04)15836-4 15051281

[B173] Smith K. G. Kamdar A. A. Stark J. M. (2019). “Lung Defenses: Intrinsic, Innate, and Adaptive” in Kendig's Disorders of the Respiratory Tract in Children (Elsevier), 120–133.e122. doi: 10.1016/B978-0-323-44887-1.00008-0

[B174] Souza M. C. Silva J. D. Padua T. A. Capelozzi V. L. Rocco P. R. Henriques M. (2013). Early and Late Acute Lung Injury and Their Association With Distal Organ Damage in Murine Malaria. Respir. Physiol. Neurobiol. 186 (1), 65–72. doi: 10.1016/j.resp.2012.12.008 23328346

[B175] Spangler W. L. Gribble D. Abildgaard C. Harrison J. (1978). Plasmodium Knowlesi Malaria in the Rhesus Monkey. Vet. Pathol. 15 (1), 83–91. doi: 10.1177/030098587801500110 415405

[B176] Spindler V. Schlegel N. Waschke J. (2010). Role of GTPases in Control of Microvascular Permeability. Cardiovasc. Res. 87 (2), 243–253. doi: 10.1093/cvr/cvq086 20299335

[B177] Srivastava K. (2014). The Role of Platelets in Malarial Acute Lung Injury and Acute Respiratory Distress Syndrome: A World of Possibilities. Acta Medica Int. 1 (2), 117–123. doi: 10.5530/ami.2014.2.16

[B178] Srivastava K. Cockburn I. A. Swaim A. Thompson L. E. Tripathi A. Fletcher C. A.. (2008). Platelet Factor 4 Mediates Inflammation in Experimental Cerebral Malaria. Cell Host Microbe 4 (2), 179–187. doi: 10.1016/j.chom.2008.07.003 18692777 PMC2603442

[B179] Ta T. H. Hisam S. Lanza M. Jiram A. I. Ismail N. Rubio J. M. (2014). First Case of a Naturally Acquired Human Infection With Plasmodium Cynomolgi. Malar. J. 13, 68. doi: 10.1186/1475-2875-13-68 24564912 PMC3937822

[B180] Taoufiq Z. Gay F. Balvanyos J. Ciceron L. Tefit M. Lechat P.. (2008). Rho Kinase Inhibition in Severe Malaria: Thwarting Parasite-Induced Collateral Damage to Endothelia. J. Infect. Dis. 197 (7), 1062–1073. doi: 10.1086/528988 18419473

[B181] Taoufiq Z. Pino P. N'Dilimabaka N. Arrouss I. Assi S. Soubrier F.. (2011). Atorvastatin Prevents Plasmodium Falciparum Cytoadherence and Endothelial Damage. Malar. J. 10, 52. doi: 10.1186/1475-2875-10-52 21356073 PMC3056843

[B182] Taylor T. E. Borgstein A. Molyneux M. E. (1993). Acid-Base Status in Paediatric Plasmodium Falciparum Malaria. Q. J. Med. 86 (2), 99–109. 8464997

[B183] Taylor W. R. J. Hanson J. Turner G. D. H. White N. J. Dondorp A. M. (2012). Respiratory Manifestations of Malaria. Chest 142 (2), 492–505. doi: 10.1378/chest.11-2655 22871759

[B184] Thachil J. (2017). Platelets and Infections in the Resource-Limited Countries With a Focus on Malaria and Viral Haemorrhagic Fevers. Br. J. Haematol. 177 (6), 960–970. doi: 10.1111/bjh.14582 28295179

[B185] Togbe D. de Sousa P. L. Fauconnier M. Boissay V. Fick L. Scheu S.. (2008). Both Functional LTbeta Receptor and TNF Receptor 2 Are Required for the Development of Experimental Cerebral Malaria. PloS One 3 (7), e2608. doi: 10.1371/journal.pone.0002608 18612394 PMC2442868

[B186] Tong M. J. Ballantine T. V. Youel D. B. (1972). Pulmonary Function Studies in Plasmodium Falciparum Malaria. Am. Rev. Respir. Dis. 106 (1), 23–29. doi: 10.1164/arrd.1972.106.1.23 4556536

[B187] Turner L. Lavstsen T. Berger S. S. Wang C. W. Petersen J. E. Avril M.. (2013). Severe Malaria Is Associated With Parasite Binding to Endothelial Protein C Receptor. Nature 498 (7455), 502–505. doi: 10.1038/nature12216 23739325 PMC3870021

[B188] Tutkun L. Iritas S. B. Deniz S. Oztan O. Abusoglu S. Unlu A.. (2019). TNF-Alpha and IL-6 as Biomarkers of Impaired Lung Functions in Dimethylacetamide Exposure. J. Med. Biochem. 38 (3), 276–283. doi: 10.2478/jomb-2018-0040 31156337 PMC6534953

[B189] Valecha N. Pinto R. G. Turner G. D. Kumar A. Rodrigues S. Dubhashi N. G.. (2009). Histopathology of Fatal Respiratory Distress Caused by Plasmodium Vivax Malaria. Am. J. Trop. Med. Hyg. 81 (5), 758–762. doi: 10.4269/ajtmh.2009.09-0348 19861606

[B190] Val F. Machado K. Barbosa L. Salinas J. L. Siqueira A. M. Costa Alecrim M. G.. (2017). Respiratory Complications of Plasmodium Vivax Malaria: Systematic Review and Meta-Analysis. Am. J. Trop. Med. Hyg. 97 (3), 733–743. doi: 10.4269/ajtmh.17-0131 28722625 PMC5590608

[B191] Van den Steen P. E. Deroost K. Deckers J. Van Herck E. Struyf S. Opdenakker G. (2013). Pathogenesis of Malaria-Associated Acute Respiratory Distress Syndrome. Trends Parasitol. 29 (7), 346–358. doi: 10.1016/j.pt.2013.04.006 23742967

[B192] Van den Steen P. E. Geurts N. Deroost K. Van Aelst I. Verhenne S. Heremans H.. (2010). Immunopathology and Dexamethasone Therapy in a New Model for Malaria-Associated Acute Respiratory Distress Syndrome. Am. J. Respir. Crit. Care Med. 181 (9), 957–968. doi: 10.1164/rccm.200905-0786OC 20093644

[B193] Vandermosten L. Pham T. T. Possemiers H. Knoops S. Van Herck E. Deckers J.. (2018). Experimental Malaria-Associated Acute Respiratory Distress Syndrome Is Dependent on the Parasite-Host Combination and Coincides With Normocyte Invasion. Malar. J. 17 (1), 102. doi: 10.1186/s12936-018-2251-3 29506544 PMC5839036

[B194] van der Pluijm R. W. Amaratunga C. Dhorda M. Dondorp A. M. (2021). Triple Artemisinin-Based Combination Therapies for Malaria - A New Paradigm? Trends Parasitol. 37 (1), 15–24. doi: 10.1016/j.pt.2020.09.011 33060063

[B195] van der Poll T. (2013). The Endothelial Protein C Receptor and Malaria. Blood 122 (5), 624–625. doi: 10.1182/blood-2013-06-508531 23908442

[B196] Vieira-de-Abreu A. Campbell R. A. Weyrich A. S. Zimmerman G. A. (2012). Platelets: Versatile Effector Cells in Hemostasis, Inflammation, and the Immune Continuum. Semin. Immunopathol. 34 (1), 5–30. doi: 10.1007/s00281-011-0286-4 21818701 PMC4334392

[B197] Villegas-Mendez A. de Souza J. B. Murungi L. Hafalla J. C. Shaw T. N. Greig R.. (2011). Heterogeneous and Tissue-Specific Regulation of Effector T Cell Responses by IFN-Gamma During Plasmodium Berghei ANKA Infection. J. Immunol. 187 (6), 2885–2897. doi: 10.4049/jimmunol.1100241 21880980 PMC3173971

[B198] Vincent P. A. Xiao K. Buckley K. M. Kowalczyk A. P. (2004). VE-Cadherin: Adhesion at Arm's Length. Am. J. Physiol. Cell Physiol. 286 (5), C987–C997. doi: 10.1152/ajpcell.00522.2003 15075197

[B199] Ware L. B. (2006). Pathophysiology of Acute Lung Injury and the Acute Respiratory Distress Syndrome. Semin. Respir. Crit. Care Med. 27 (4), 337–349. doi: 10.1055/s-2006-948288 16909368

[B200] Wassmer S. C. Lepolard C. Traore B. Pouvelle B. Gysin J. Grau G. E. (2004). Platelets Reorient Plasmodium Falciparum-Infected Erythrocyte Cytoadhesion to Activated Endothelial Cells. J. Infect. Dis. 189 (2), 180–189. doi: 10.1086/380761 14722881

[B201] Wei H. Jin C. Peng A. Xie H. Xie S. Feng Y.. (2021). Characterization of gammadeltaT Cells in Lung of Plasmodium Yoelii-Infected C57BL/6 Mice. Malar. J. 20 (1), 89. doi: 10.1186/s12936-021-03619-z 33588839 PMC7885449

[B202] Weiss M. L. Kubat K. (1983). Plasmodium berghei: A Mouse Model for the "Sudden Death" and "Malarial Lung" Syndromes. Exp. Parasitol. 56 (1), 143–151. doi: 10.1016/0014-4894(83)90105-4 6223835

[B203] West N. R. (2019). Coordination of Immune-Stroma Crosstalk by IL-6 Family Cytokines. Front. Immunol. 10, 1093. doi: 10.3389/fimmu.2019.01093 31156640 PMC6529849

[B204] Wettschureck N. Strilic B. Offermanns S. (2019). Passing the Vascular Barrier: Endothelial Signaling Processes Controlling Extravasation. Physiol. Rev. 99 (3), 1467–1525. doi: 10.1152/physrev.00037.2018 31140373

[B205] WHO. (2021). World Malaria Report 2021 (Geneva: World Health Organization).

[B206] William T. Menon J. Rajahram G. Chan L. Ma G. Donaldson S.. (2011). Severe Plasmodium Knowlesi Malaria in a Tertiary Care Hospital, Sabah, Malaysia. Emerg. Infect. Dis. 17 (7), 1248–1255. doi: 10.3201/eid1707.101017 21762579 PMC3381373

[B207] Xie R. F. Hu P. Wang Z. C. Yang J. Yang Y. M. Gao L.. (2015). Platelet-Derived Microparticles Induce Polymorphonuclear Leukocyte-Mediated Damage of Human Pulmonary Microvascular Endothelial Cells. Transfusion 55 (5), 1051–1057. doi: 10.1111/trf.12952 25565376

[B208] Yeo T. W. Lampah D. A. Gitawati R. Tjitra E. Kenangalem E. Piera K.. (2008). Angiopoietin-2 Is Associated With Decreased Endothelial Nitric Oxide and Poor Clinical Outcome in Severe Falciparum Malaria. Proc. Natl. Acad. Sci. U. S. A. 105 (44), 17097–17102. doi: 10.1073/pnas.0805782105 18957536 PMC2575222

[B209] Zang-Edou E. S. Bisvigou U. Taoufiq Z. Lekoulou F. Lekana-Douki J. B. Traore Y.. (2010). Inhibition of Plasmodium Falciparum Field Isolates-Mediated Endothelial Cell Apoptosis by Fasudil: Therapeutic Implications for Severe Malaria. PloS One 5 (10), e13221. doi: 10.1371/journal.pone.0013221 20949056 PMC2951358

[B210] Zhao T. Liu M. Gu C. Wang X. Wang Y. (2014). Activation of C-Src Tyrosine Kinase Mediated the Degradation of Occludin in Ventilator-Induced Lung Injury. Respir. Res. 15, 158. doi: 10.1186/s12931-014-0158-2 25471013 PMC4262993

[B211] Zielinska K. A. de Cauwer L. Knoops S. van der Molen K. Sneyers A. Thommis J.. (2017). Plasmodium Berghei NK65 in Combination With IFN-Gamma Induces Endothelial Glucocorticoid Resistance *via* Sustained Activation of P38 and JNK. Front. Immunol. 8, 1199. doi: 10.3389/fimmu.2017.01199 29033931 PMC5625030

